# Age-Related Patterns of Type II Interferon Immunity: Implications for Intramacrophagic Infections and MSMD Diagnosis During Childhood

**DOI:** 10.1007/s10875-025-01955-2

**Published:** 2025-12-26

**Authors:** Yiyi Luo, Guillermo Argüello, Daniel Acevedo, Cristina Jou, Anna Codina, Jesús Márquez, Alexandru Vlagea, Sara Peiró, Víctor Bolaño, Aina Freixedas, Angela Deyà-Martínez, Ana García-García, Celia Martí-Castellote, Manel Juan, Ana Esteve-Solé, Laia Alsina

**Affiliations:** 1 Study Group for Immune Dysfunction Diseases in Children (GEMDIP), Institut de Recerca Sant Joan de Déu, Barcelona (IRSJD), Barcelona, EU, Spain; 2 Pediatric Allergy and Clinical Immunology Department, Clinical Immunology and Primary Immunodeficiencies Unit, Hospital Sant Joan de Déu, Barcelona, EU, Spain; 3 Clinical Immunology Unit, Hospital Sant Joan de Déu-Hospital Clínic, Barcelona, EU, Spain; 4 MedSavana SL, Madrid, Spain; 5 Faculty of Computer Science, Multimedia and Telecommunications, Universitat Oberta de Catalunya, Barcelona, Spain; 6 Biobank and Pathology department. Institut de Recerca Sant Joan de Déu, Hospital Sant Joan de Déu, CIBERER ISCIII, Barcelona, 08950 Spain; 7 Immunology Department, Biomedic Diagnostic Center (CDB), Hospital Clínic of Barcelona, Barcelona, Spain; 8 Department of Surgery and Surgical Specializations, Faculty of Medicine and Health Sciences, University of Barcelona, Barcelona, EU, Spain

**Keywords:** Interferon-gamma, Chemokine CXCL10, STAT1 transcription factor, T-lymphocytes, CFSE, Primary immunodeficiency

## Abstract

Type II interferon (IFN) immunity is crucial for controlling intramacrophagic infections, driven by the interaction between innate immunity (macrophage-derived IL-12) and adaptive immunity (Th-derived IFN-γ). This study examines the maturation of type II IFN immunity in 55 healthy children (ages 1–18) to enable proper identification of deficiencies as part of the diagnostic evaluation of Mendelian Susceptibility to Mycobacterial Diseases (MSMD). The IL-12/IFN-γ axis was assessed through: (1) cytokine production after mycobacterial stimulation (Luminex and ELISA for IFN-γ, IL-12p70, TNF, CXCL10, IL-1RA, IL-10, IL-1β and IL-6), (2) IFN-γR1/R2 expression on monocytes, and (3) STAT1 phosphorylation/dephosphorylation. T cell maturation (primary IFN-γ source) was evaluated via immunophenotyping (naïve/memory/activated, Th1; Th2; Th17; Th1/17; Tfh) and proliferation assays. Main findings: (1) stable expression/production of key components of the IL-12/IFN-γ axis (IFN-γ, IL-12, TNF, IFN-γR1/2, and STAT1 activity) across ages confirming the stability of innate immune function throughout childhood; (2) increasing responses to IFN-γ with age reflected by increased CXCL10 production, and increase in the IFN-γ counter-acting anti-inflammatory cytokines (IL-10, IL-1RA); and (3) progressive T cell maturation, including Th1, Th17 and Th1/17 subsets, with significant milestones between 6 and 8.6 years, while T cell proliferative capacity remained stable. These observations highlight the stability of IL-12/IFN-γ axis innate components with age, accompanied by enhanced downstream IFN-γ signaling, aligning with the maturation of Th cell compartment. These underscore the limited benefit of age-specific controls in the evaluation of IL-12/IFN-γ axis in MSMD diagnosis, while emphasizing the importance of T cell maturation in the overall type II IFN immunity.

**Graphical Abstract:**

**Figure:**
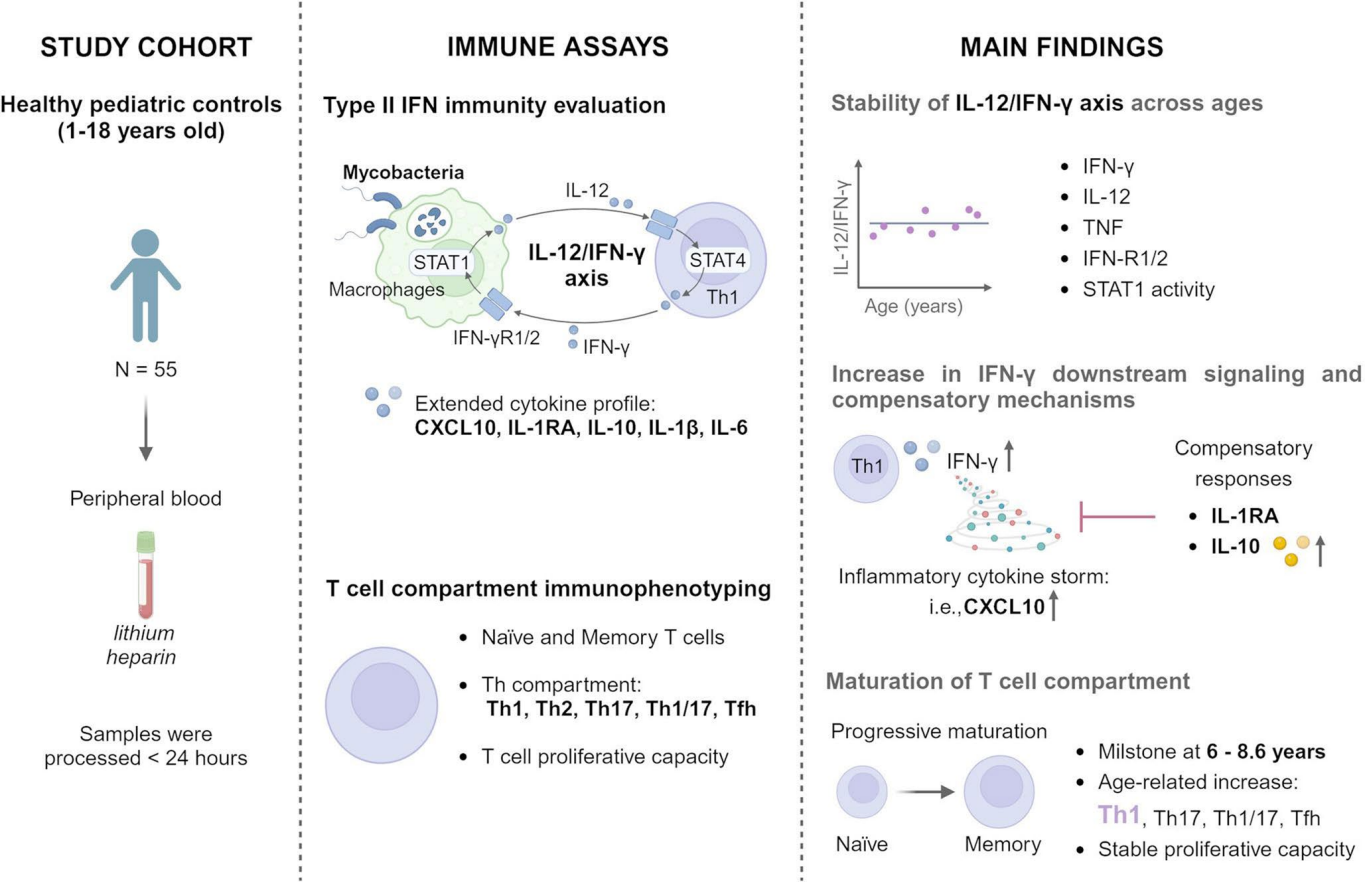
Created with Biorender.com

**Supplementary Information:**

The online version contains supplementary material available at 10.1007/s10875-025-01955-2.

## Introduction

Inborn errors of immunity (IEI) are a group of genetic disorders caused by mutations in genes affecting immune development, regulation, or function [1]. They often present during infancy and childhood, leading to increased susceptibility to infections, autoimmunity, autoinflammation, allergy, bone marrow failure, and/or malignancy [2, 3]. Simultaneously, the immune system matures progressively from infancy to adulthood, with stage-specific characteristics and vulnerabilities [4, 5]. The overlap can blur the distinction between normal age-related immune variations and true IEI [4–6]. For example, neonatal immunity favors regulatory responses to limit inflammation from microbial colonization at birth [4, 7], characterized by enhanced polarization T helper (Th) 2 cell and alternatively activated macrophages, alongside downregulation of Th1 cells and classically activated macrophages [4, 5, 8–10]. Consequently, it limits pro-inflammatory cytokines, such as interferon (IFN)-γ, tumor necrosis factor (TNF), and interleukin (IL)−17 [9, 10], while enhancing levels of immunosuppressive cytokines like IL-10 [11], which offers protection from inflammation but raising susceptibility to intramacrophagic infections and allergy in early life [12]. The fine equilibrium underscores the complexity of immune development in early life [4].

The European Society for Immunodeficiencies (ESID) recommends immune assays as part of the diagnostic work-up for suspected IEI [13], emphasizing the integration of laboratory results with clinical and genetic findings [14]. Currently, reference values for selected immune parameters—such as lymphocyte subsets and serum biomarkers—are widely utilized in diagnostic evaluations [15–21]. However, these reference values do not cover the full spectrum of immune assessments required for diagnosing IEI and are often limited in age-specific stratification [15, 16]. In addition, there is a lack of standardization in defining lymphocyte subsets [17–19] and inconsistencies in functional assays such as cytokine production and proliferation [6, 20–23]. When pediatric-specific values are unavailable, results are often compared to adult reference ranges [24, 25], potentially overlooking developmental patterns of the immune system [4], which is especially relevant in diseases like Mendelian Susceptibility to Mycobacterial Disease (MSMD, OMIM 209950) [24, 25].

MSMD is a rare IEI (1/50,000 individuals) caused by genetic alterations in Type II IFN immunity, which broadly encompasses the responses mediated by IFN-γ [26–30]. Patients exhibit a selective predisposition to severe diseases caused by weakly virulent mycobacteria, including *Mycobacterium bovis* Bacille Calmette-Guérin (BCG) vaccines, as well as more virulent *Mycobacterium tuberculosis* in otherwise healthy individuals [31, 32]. Less frequently, patients are also vulnerable to other intracellular pathogens, such as *Salmonella*, and related bacteria, fungi (i.e., *C.albicans*), and parasites (i.e., *Leishmania* species) [30]. MSMD typically presents in early life and can be life-threatening [26–29, 33], prompt diagnosis is critical. Differential diagnosis includes severe combined immunodeficiency, chronic granulomatous disease, and other IEIs [34–37]. To date, 21 genes involved in Type II IFN immunity have been implicated in MSMD, 18 autosomal (*IFNGR1*,* IFNGR2*,* IFNG*,* STAT1*,* JAK1*,* IRF8*,* IRF1*,* SPPL2A*,* TYK2*,* IL12B*,* IL12RB1*,* IL12RB2*,* IL23R*,* TBX21*,* RORC*,* ZNFX1*,* ISG15* and *USP18*) and three X-linked (*CYBB*,* NEMO* and *MCTS1*) [6, 30, 33, 38]. Despite expanded access to next-generation sequencing, only about 50% of patients with an MSMD-like phenotype receive a confirmed genetic diagnosis [39, 40]. Thus, current diagnostic guidelines recommend combining both genetic analysis with functional laboratory immune assays [6, 13, 24, 41] including evaluation of the Type II IFN immunity [6].

Type II IFN immunity plays a central role controlling intramacrophagic infections [42] through interactions between innate (macrophage-derived IL-12) and adaptive (Th-derived IFN-γ) arms of immunity [43]. Upon mycobacterial challenge, macrophages release IL-12, which stimulates IFN-γ production by Th1 and NK cells [6, 44, 45]. IFN-γ further promotes Th1 differentiation, proliferation, and macrophage activation [46, 47]. Th1/NK-derived IFN-γ binds to IFN-γ receptor (IFN-γR)−1/IFN-γR−2 on macrophages, inducing the phosphorylation of signal transducer and activator of transcription (pSTAT)−1, resulting in the transcription of genes for macrophage activation (microbicidal effect), including tumor necrosis factor (TNF) with autocrine functions that enhance macrophage activation [6]. This immune crosstalk establishes a feedback loop known as the IL-12/IFN-γ axis (Figure S1).

Additional cytokines also significantly contribute to shaping and regulating the axis network’s responses [5, 48, 49]. Chemokine (C-X-C motif) ligand 10, also known as IFN-γ induced protein (IP)−10, is a potent pro-inflammatory cytokine produced by monocytes (among other cells) in response to IFN-γ [48]. CXCL10 recruits activated T cells, NK cells, and to a lesser extent, macrophages and dendritic cells to sites of infection [50], and enhances Th1 polarization by acting on CXCR3 + naïve T cells—establishing a positive feedback loop between IFN-γ-producing Th1 cells and CXCL10-producing resident cells [51]. CXCL10 is also a reliable diagnostic and treatment-response biomarker for tuberculosis in children and adults [52–54]. Conversely, IFN-γ and IL-12 also induce anti-inflammatory cytokines to balance inflammation [5, 49]. IL-1 receptor antagonist (IL-1RA) inhibits IL-1 signaling induced by IFN-γ, contributing to the control of excessive inflammation [49]. Similarly, IL-10, primarily produced by regulatory T and B cells, acts as a negative regulator of type II IFN immunity [5]. These regulatory mechanisms integrate with the IL-12/IFN-γ axis to ensure a balanced immune response, preventing tissue damage while maintaining effective pathogen control [4].

A clearer understanding of age-related development of type II IFN immunity is essential to distinguish physiological immaturity from pathological immune dysfunction, particularly in children with suspected MSMD. Current immunoassays assess the integrity of the IL-12/IFN-γ axis using a range of laboratory tests, including [6]: (1) IFN-γ level in plasma (baseline level) [55], (2) cytokine secretion upon stimulus/mycobacterial challenge, (3) expression of IFN-γR-1 and IFN-γR−2 on monocytes, (4) IL-12 receptor (IL-12R)-β1 expression on T cells, and 4) pSTAT1 in response to IFN-γ [6]. Since IFN-γ is mainly produced by Th1 [47], assessing T cell phenotypes and functions is essential when fully evaluating Type II IFN immunity. Additionally, measuring downstream cytokines such as CXCL10 provides complementary insight into the functional responsiveness and signaling output of the IL-12/IFN-γ axis [23, 46, 47].

This study aimed to characterize the maturation of type II IFN immunity across childhood using a multi-parametric approach that integrates cytokine production, receptor expression, intracellular signaling, and T cell phenotyping, to support more accurate clinical evaluation of pediatric MSMD.

## Methods

Sample collection and immune assay methods are graphically represented in Figure S1.

### Subjects and Sample Collection

Peripheral heparinized whole blood was collected in lithium heparin vacutainer tubes (BD, Cat 367885) and processed within 24 h. Healthy pediatric controls were recruited from patients undergoing elective surgeries (e.g., ear, nose, throat, or phimosis surgeries) at Hospital Sant Joan de Déu. Inclusion criteria: age 1–18 years, informed consent/assent. Exclusion criteria: known chromosomal, oncological, hematological, cardiac, or immune conditions, and active infections at sampling. The study adhered to General Health Law guidelines (Ley General de Sanidad, 1986) and received ethical approval (PIC-129-18) from the Hospital Sant Joan de Déu Ethics Committee.

### IL-12/IFN-γ Axis Activity in Response to Mycobacterial Stimulus

Heparinized whole blood was cultured as a gold standard method to assess the cytokine profile [56, 57]. Cells were diluted 1:2 in RPMI (Gibco, Grand Island, NY, USA) with supplements [10% heat-inactivated fetal calf serum (Sigma-Aldrich, St. Louis, MO, USA), 1 µg/ml penicillin, and 1 µg/ml streptomycin (Invitrogen, Grand Island, NY, USA)] and incubated under six stimulation conditions: (1) baseline, (2) BCG (M. bovis BCG, Pasteur substrain), (3) BCG plus human recombinant (hr) IL-12p70 (20 ng/ml, Miltenyi Biotec, Germany), (4) BCG plus hrIFN-γ (5,000 IU/ml; Imukin, Boehringer Ingelheim, Germany), (5) phorbol myristate acetate (PMA, 50ng/mL, Sigma-Aldrich, St. Louis, USA) plus ionomycin (1ug/mL, Sigma-Aldrich, St. Louis, USA), and (6) TNF (10 µg/mL, Milteny Biotec, Bergisch Gladbach, Germany). Condition 5 served as a positive control, and condition 6 was used to evaluate IL-10 production for diagnosing genetic defects in the NF-κB essential modulator deficiency [6].

Baseline IFN-γ levels in plasma and stimulated production from culture supernatants were assessed using enzyme-linked immunosorbent assay (ELISA, Invitrogen, Grand Island, NY, USA) after 48 h. Additionally, cytokine levels - IFN-γ, IL-12p70, TNF, CXCL10, IL-1RA, IL-10, IL-1β and IL-6 - were quantified from culture supernatants using Luminex assay (Millipore, Billerica, MA, USA) after 18 h. Stimulation ratios (SR: stimulated condition/basal condition) and co-stimulation ratios (co-SR: BCG plus hrIFN-γ or hrIL-12/BCG) were then calculated.

### Multiparameter Flow Cytometry and Proliferation Assays

We used multiparameter flow cytometry to evaluate various immunological parameters, including IFN-γR1/IFN-γR2 expression, pSTAT1/dephosphorylation STAT1 (dSTAT1), T cell immunophenotyping and proliferation. IFN-γR1/IFN-γR2 expression were analyzed by staining heparinized whole blood with IFN-γR1-PE (BD; Cat: 558937) and IFN-γR2-APC (R&D Systems; Cat: FAB773A). pSTAT1/dSTAT1 were evaluated in monocytes (CD14-APC; BD; Cat: 555399) following stimulation with hrIFN-γ and IFN-α (Pegasys, Roche, Paris, France) in the presence or not of staurosporine (Sigma-Aldrich). For both IFN-γR1/IFN-γR2 expression and STAT1 activity we analyzed the percentage (%) and the mean fluorescence intensity (MFI). T cell subsets, including naïve, memory, activated, Th1-Th2-Th17-Th1/17-Tfh cells, were identified using monoclonal antibodies (mAbs; details in Table S1). T cell proliferation was assessed in peripheral blood mononuclear cells (PBMC) cultured with mitogens [phytohemaglutinin A (5 µg/mL; PHA, Sigma, St. Louis, MO, USA), pokeweed mitogen (2 µg/mL; PWM; Sigma, St. Louis, MO, USA) and Concanavalin A (2 µg/mL; ConA, Sigma, St. Louis, MO, USA)] and labeled with carboxifluorescein diacetate succinimidyl ester (CFSE, Invitrogen, Grand Island, NY, USA) [23]; after seven days, cells were stained with CD19-PE-Cy7 (BD; Cat: 557835), CD3-APC-H7 (BD; Cat: 641415), and CD4-FITC (BD; Cat: 345768) to measure division index (DI) and proliferation index (PI).

The supplementary material outlines the methods and materials supporting this study, including sample processing protocols, reagent compositions, and flow cytometry specifications. It details mAb panels (Table S1), functional test reagents (Table S2), gating strategies (Table S3, Figure S2−5), and specifies FACSCanto-II, FlowJo v.10, Luminex, and ELISA settings to ensure reproducibility and cytokine quantification accuracy.

### Statistical Analysis

Categorical variables are reported as frequency (n) and percentage (%), and non-normally distributed continuous variables as median (Mdn) and interquartile range (IQR). Group comparisons were made using the Kruskal-Wallis test (*p* < 0.05). Correlations were assessed with Spearman’s rank coefficient, with values ranging from − 1 (negative) to + 1 (positive) [58]. Statistical analyses were performed in Python 3.12, and graphs were created using Python and Prism 7.04 (GraphPad, La Jolla, CA). Further details are in the Supplementary Material.

## Results

The study cohort comprised 55 heparinized whole blood samples from healthy pediatric controls (1 to 18 years): 8 samples from children aged 1–3 years (7 males, 1 female), 5 samples aged > 3–5 years (all males), 9 samples aged > 5–7 years (7 males, 2 females), 12 samples aged > 7–10 years (8 males, 4 females), 5 samples aged > 10–14 years (1 male, 4 females), and 6 samples aged > 14–18 years (4 males, 2 females). All donors were of White-European origin.

### Age-Dependent Variations in the IL-12/IFN-γ Axis in Response to BCG: Consistent Production of IFN-γ, IL-12, TNF, and Increased CXCL10, IL-10, and IL-1RA

**Fig. 1:**
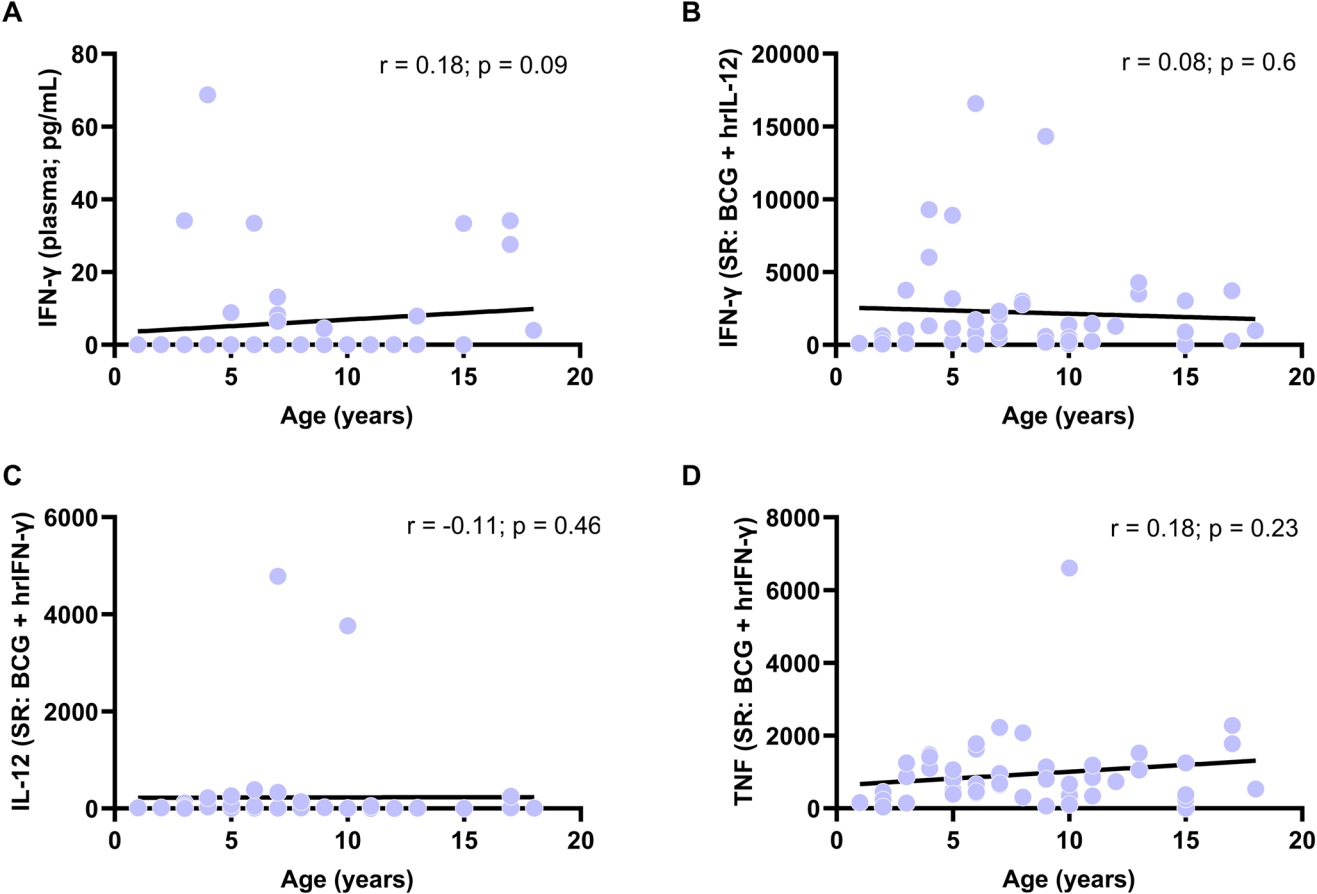
Levels of IFN-γ, IL-12, and TNF in a healthy pediatric population (n = 55; ages 1–18 years) correlated with age. A Basal plasma IFN-γ levels (pg/mL) measured by ELISA. B IFN-γ levels (stimulation ratio: SR, calculated as stimulated/basal condition) in response to BCG plus hrIL-12. C-D IL-12 and TNF levels (SR) in response to BCG plus hrIFN-γ. B-D Cytokine levels were analyzed by Luminex. No significant correlations were observed between age and the levels of these three cytokines suggesting stability of IL-12/ IFN-γ axis. Statistical significance p < 0.05; Spearman correlation (r): low association 0.1-0.3; moderate positive association between 0.3-0.5; and strong positive association 0.5-1.

Cytokine production in response to BCG was assessed using both ELISA and Luminex, following MSMD diagnostic protocols [6, 13]. Descriptive data are depicted in Table 1 (IFN-γ by ELISA) and Table 2 (cytokines by Luminex). Correlations of SR and co-SR in Figure S6 and Figure S7. Cytokine profiles in baseline condition, and in response to PMA/ionomycin and TNF are shown in Table S4A-B. Baseline plasma IFN-γ levels showed a weak, non-significant correlation with age (*r* = 0.18; *p* = 0.09; Fig. 1A; Table 1). Similarly, IFN-γ levels in response to BCG plus hrIL-12 were non-significant [*r* = −0.18; *p* = 0.23 by ELISA (Figure S6) and *r* = 0.08; *p* = 0.6 by Luminex (Fig. 1B)]. In response to BCG plus hrIFN-γ, IL-12 (*r* = −0.11; *p* = 0.46) and TNF (*r* = 0.18; *p* = 0.23) also showed no significant correlation with age (Fig. 1C and D). Additionally, no significant age-related variations were observed for IL-1β or IL-6 levels (*r* < 0.2 for SR; *p* >0.05; Figure S7A and Table 2).

**Table 1:** Baseline levels of IFN-γ (pg/mL) in plasma and in response to mycobacterial challenge from 1–18 years old (yo). Values are presented as median and interquartile range (Q1–Q3)

Stimulation condition	1–3 yo (*N* = 9)	> 3–5 yo (*N* = 6)	> 5–7 yo (*N* = 9)	> 7–10 yo (*N* = 14)	> 10–14 yo (*N* = 6)	> 14–18 yo (*N* = 8)	Correlation with age (*r*)	*p*-value
Plasma	0.0 (0.0–0.0)	0.0 (0.0–0.0)	0.0 (0.0–8.8.0.8)	0.0 (0.0–0.0)	0.0 (0.0–0.0)	15.8 (1.0–31.9.0.9)	0.18	0.23
SR: BCG	232.8 (113.8–539.1.8.1)	355.4 (207.3–521.3.3.3)	498.3 (197.2–568.0)	161.4 (78.2–840.3.2.3)	112.3 (57.0–407.5.0.5)	326.0 (295.3–773.7.3.7)	−0.01	0.93
SR: BCG + IL-12	12072.3 (7042.1–34227.0.1.0)	16876.2 (12017.3–20706.4.3.4)	17857.8 (9347.2–34417.7.2.7)	14173.1 (3643.0–22821.8.0.8)	12551.6 (7573.0–17662.5.0.5)	11858.4 (8589.3–16865.7.3.7)	−0.18	0.23
SR: PMA + Ionomycin	37759.3 (35288.5–41540.0.5.0)	52225.9 (45106.9–63311.4.9.4)	48454.5 (38035.8–62179.4.8.4)	56729.2 (39164.4–67188.4.4.4)	52165.5 (28925.1–72507.1.1.1)	64205.4 (48021.7–76122.1.7.1)	0.33	0.03
Co-SR: BCG + IL-12	100.9 (92.5–104.3.5.3)	35.5 (31.0–66.1.0.1)	48.7 (36.1–62.2)	78.0 (42.7–120.6.7.6)	78.4 (39.9–125.0)	29.0 (21.7–41.6)	−0.23	0.18

Abbreviations: BCG: SR: stimulation ratio (stimulated condition/basal condition); Co-SR (BCG plus IL-12/BCG). Spearman correlation (r): low association 0.1–0.3; moderate positive association between 0.3–0.5; and strong positive association 0.5–1.5. Statistical significance *p* < 0.05

**Table 2:** Cytokine levels - IFN-γ, IL-12p70, TNF, CXCL10, IL-RA, IL-10, IL-1β and IL-6 - in response to mycobacterial challenge from 1–18 years old (yo). Values are presented as median and interquartile range (Q1–Q3)

Cytokine	Stimulation condition	1–3 yo (*N* = 9)	> 3-5yo (*N* = 6)	> 5-7yo (*N* = 9)	> 7-10yo (*N* = 14)	> 10-14yo (*N* = 6)	> 14-18yo (*N* = 8)	Correlation with age (*r*)	*p*-value
**IFN-γ**	SR: BCG	2.6 (1.7–5.6)	8.7 (3.2–8.7)	6.9 (4.1–20.5)	7.0 (2.2–10.9)	2.6 (2.3–4.0.3.0)	6.0 (1.4–10.6)	0.03	0.85
	SR: BCG + IL-12	291.0 (88.8–813.7.8.7)	2238.9 (463.4–5304.4.4.4)	849.6 (497.7–1677.0)	910.3 (485.8–2304.5.8.5)	1454.0 (1318.4–2985.3.4.3)	881.7 (170.0–1998.0.0.0)	0.08	0.60
	Co-SR: BCG + IL-12	124.7 (51.5–239.4.5.4)	179.8 (138.8–324.2.8.2)	109.0 (84.8–122.8.8.8)	180.8 (104.7–345.5.7.5)	530.4 (302.9–618.6.9.6)	65.3 (53.1–201.2.1.2)	0.07	0.61
**IL-12p70**	SR: BCG	1.0 (1.0–1.2.0.2)	0.9 (0.8–1.2)	1.0 (0.9–1.2)	1.3 (1.0–1.7.0.7)	1.4 (1.1–1.6)	1.0 (0.7–2.0.7.0)	0.18	0.22
	SR: BCG + IFN-γ	11.7 (3.3–25.0)	52.2 (10.2–184.2.2.2)	14.0 (7.8–45.7)	19.8 (10.5–139.3.5.3)	7.1 (3.3–8.0.3.0)	8.7 (3.1–15.1)	−0.11	0.46
	Co-SR: BCG + IFN-γ	7.9 (3.6–11.5)	30.5 (9.8–216.5.8.5)	19.2 (8.4–74.2)	19.1 (5.9–95.7)	5.0 (2.8–8.8)	7.2 (3.9–21.0)	−0.14	0.33
**TNF**	SR: BCG	176.3 (25.6–465.4.6.4)	492.6 (301.0–682.3.0.3)	498.0 (327.8–714.4.8.4)	358.6 (204.9–649.5.9.5)	395.4 (298.0–539.3.0.3)	581.2 (345.2–699.8.2.8)	0.13	0.39
	SR: BCG + IFN-γ	224.1 (96.7–657.4.7.4)	957.2 (592.1–1340.7.1.7)	656.3 (457.3–1061.0)	681.2 (346.9–1143.0)	950.0 (763.8–1154.8.8.8)	533.0 (317.9–1512.7.9.7)	0.18	0.23
	Co-SR: BCG + IL-12	1.1 (1.0–1.5.0.5)	1.4 (1.1–1.5)	1.2 (1.1–1.5)	1.5 (1.1–2.0.1.0)	1.9 (1.5–2.9)	1.1 (0.9–1.3)	0.01	0.94
	Co-SR: BCG + IFN-γ	1.5 (1.3–2.0.3.0)	1.7 (1.2–2.0.2.0)	1.5 (1.3–1.7)	2.0 (1.2–3.2)	1.9 (1.6–3.4)	1.4 (0.9–2.4)	0.07	0.63
**CXCL10**	SR: BCG	1.0 (0.9–1.3)	1.1 (1.1–1.3)	2.0 (1.2–5.8)	1.3 (1.1–1.6)	1.4 (1.2–1.5)	1.2 (1.1–1.5)	0.19	0.19
	SR: BCG + IFN-γ	29.6 (19.6–37.2)	56.7 (35.9–69.8)	41.5 (29.8–78.8)	64.8 (45.2–99.0)	67.9 (59.7–83.8)	71.4 (45.9–75.4)	0.31	0.03
	Co-SR: BCG + IFN-γ	24.2 (20.9–30.7)	46.6 (32.3–52.7)	22.1 (7.2–29.6)	53.0 (34.7–87.8)	50.9 (43.7–54.7)	45.9 (36.1–55.0)	0.28	0.06
**IL-1RA**	SR: BCG	6.0 (5.0–24.3.0.3)	11.5 (6.6–24.1)	16.5 (7.2–25.0)	37.9 (11.6–52.4)	23.2 (19.6–27.7)	18.6 (9.7–45.0)	0.26	0.08
	SR: BCG + IL-12	7.0 (3.6–15.5)	9.8 (5.6–19.7)	13.3 (5.6–16.7)	25.1 (12.7–35.5)	21.4 (20.1–26.8)	20.4 (7.2–54.4)	0.29	0.04
	SR: BCG + IFN-γ	6.1 (5.0–17.8.0.8)	10.8 (8.7–18.3)	15.1 (10.0–22.4.0.4)	24.6 (12.8–43.1)	24.8 (22.1–24.9)	18.6 (9.3–58.6)	0.27	0.07
	Co-SR: BCG + IL-12	0.8 (0.6–0.9)	0.9 (0.8–0.9)	0.8 (0.7–0.9)	0.8 (0.6–1.0.6.0)	0.9 (0.7–1.1)	0.9 (0.8–1.1)	0.16	0.28
	Co-SR: BCG + IFN-γ	1.0 (0.8–1.1)	1.0 (0.9–1.1)	0.9 (0.8–1.0.8.0)	1.0 (0.7–1.2)	0.9 (0.9–1.1)	1.0 (1.0–1.0)	−0.00	0.99
**IL-10**	SR: BCG	24.2 (8.3–74.4)	150.7 (51.5–379.2.5.2)	84.6 (33.1–247.4.1.4)	313.1 (131.0–456.5.0.5)	336.5 (213.9–457.0)	129.9 (73.8–328.8.8.8)	0.34	0.02
	SR: BCG + IL-12	17.1 (7.3–27.0)	73.5 (31.0–124.9.0.9)	22.8 (14.5–94.6)	161.7 (65.5–235.0)	199.5 (114.6–315.7.6.7)	62.8 (50.8–186.0)	0.40	0.01
	SR: BCG + IFN-γ	2.1 (1.3–2.3)	4.2 (2.0–5.4.0.4)	2.6 (1.3–8.1)	8.6 (6.1–19.5)	15.5 (5.4–28.2)	2.0 (1.9–5.7)	0.32	0.02
	Co-SR: BCG + IL-12	0.6 (0.3–0.9)	0.5 (0.4–0.6)	0.4 (0.4–0.6)	0.5 (0.4–0.6)	0.5 (0.5–0.8)	0.7 (0.5–0.8)	0.13	0.39
	Co-SR: BCG + IFN-γ	0.1 (0.0–0.2.0.2)	0.0 (0.0–0.0)	0.0 (0.0–0.0)	0.0 (0.0–0.1.0.1)	0.0 (0.0–0.1.0.1)	0.0 (0.0–0.1.0.1)	−0.14	0.33
**IL-1β**	SR: BCG	1109.0 (227.4–14434.5.4.5)	3625.8 (1291.1–14215.1.1.1)	5094.5 (1029.5–12224.5.5.5)	1788.7 (841.1–4167.6.1.6)	4427.1 (3116.1–5059.6.1.6)	6268.9 (2168.3–6669.1.3.1)	0.15	0.32
	SR: BCG + IL-12	1117.8 (521.1–10418.0.1.0)	4696.0 (1483.3–23531.2.3.2)	5376.5 (1030.7–15409.2.7.2)	3333.8 (756.6–6134.3.6.3)	9858.1 (4718.7–14974.1.7.1)	5795.3 (3042.7–7206.2.7.2)	0.15	0.30
	SR: BCG + IFN-γ	973.1 (299.6–14766.3.6.3)	5755.6 (1755.0–15263.7.0.7)	4173.9 (1024.2–20087.1.2.1)	3956.4 (728.2–4625.9.2.9)	8521.3 (4520.2–11332.0.2.0)	3262.2 (1869.4–11735.4.4.4)	0.16	0.29
	Co-SR: BCG + IL-12	1.1 (1.0–1.5.0.5)	1.3 (1.2–1.4)	1.3 (1.0–1.5.0.5)	1.3 (1.0–1.7.0.7)	1.6 (1.1–2.8)	1.0 (0.9–1.2)	0.01	0.97
	Co-SR: BCG + IFN-γ	1.0 (0.6–1.5)	1.0 (0.7–1.2)	1.1 (1.0–1.4.0.4)	1.3 (1.1–1.7)	1.5 (1.0–2.2.0.2)	0.9 (0.7–1.9)	0.13	0.40
**IL-6**	SR: BCG	649.6 (222.5–2429.2.5.2)	1547.1 (1113.4–6865.8.4.8)	2078.2 (1010.8–2704.1.8.1)	1527.0 (741.0–1776.3.0.3)	3068.7 (2904.8–4114.9.8.9)	1381.4 (923.1–4379.4.1.4)	0.18	0.23
	SR: BCG + IL-12	741.2 (235.8–2388.9.8.9)	2214.1 (1222.7–7593.6.7.6)	2333.5 (1087.6–3309.3.6.3)	1933.2 (896.8–2560.6.8.6)	3891.7 (3278.8–4607.4.8.4)	1709.8 (1342.2–4421.8.2.8)	0.22	0.14
	SR: BCG + IFN-γ	438.2 (77.8–1381.4.8.4)	1661.2 (671.8–6137.9.8.9)	1307.8 (989.0–3299.7.0.7)	989.4 (660.1–1494.1.1.1)	2789.0 (1940.3–3272.8.3.8)	358.9 (122.2–3712.2.2.2)	0.12	0.41
	Co-SR: BCG + IL-12	1.0 (1.0–1.1.0.1)	1.1 (1.0–1.3.0.3)	1.1 (1.0–1.1.0.1)	1.2 (1.0–1.5.0.5)	1.1 (1.1–1.3)	1.2 (1.0–1.7.0.7)	0.16	0.28
	Co-SR: BCG + IFN-γ	0.5 (0.2–0.9)	0.7 (0.5–0.8)	0.9 (0.9–1.0.9.0)	0.8 (0.6–0.9)	0.8 (0.6–1.0.6.0)	0.6 (0.2–0.9)	−0.05	0.75

Abbreviations: BCG: Bacillus Calmette-Guérin; SR: stimulation ratio (stimulated condition/basal condition); Co-SR (BCG plus IL-12 or IFN-γ/BCG). Spearman correlation (r): low association 0.1–0.3; moderate positive association between 0.3–0.5; and strong positive association 0.5–1.5. Statistical significance *p* < 0.05

**Fig. 2:**
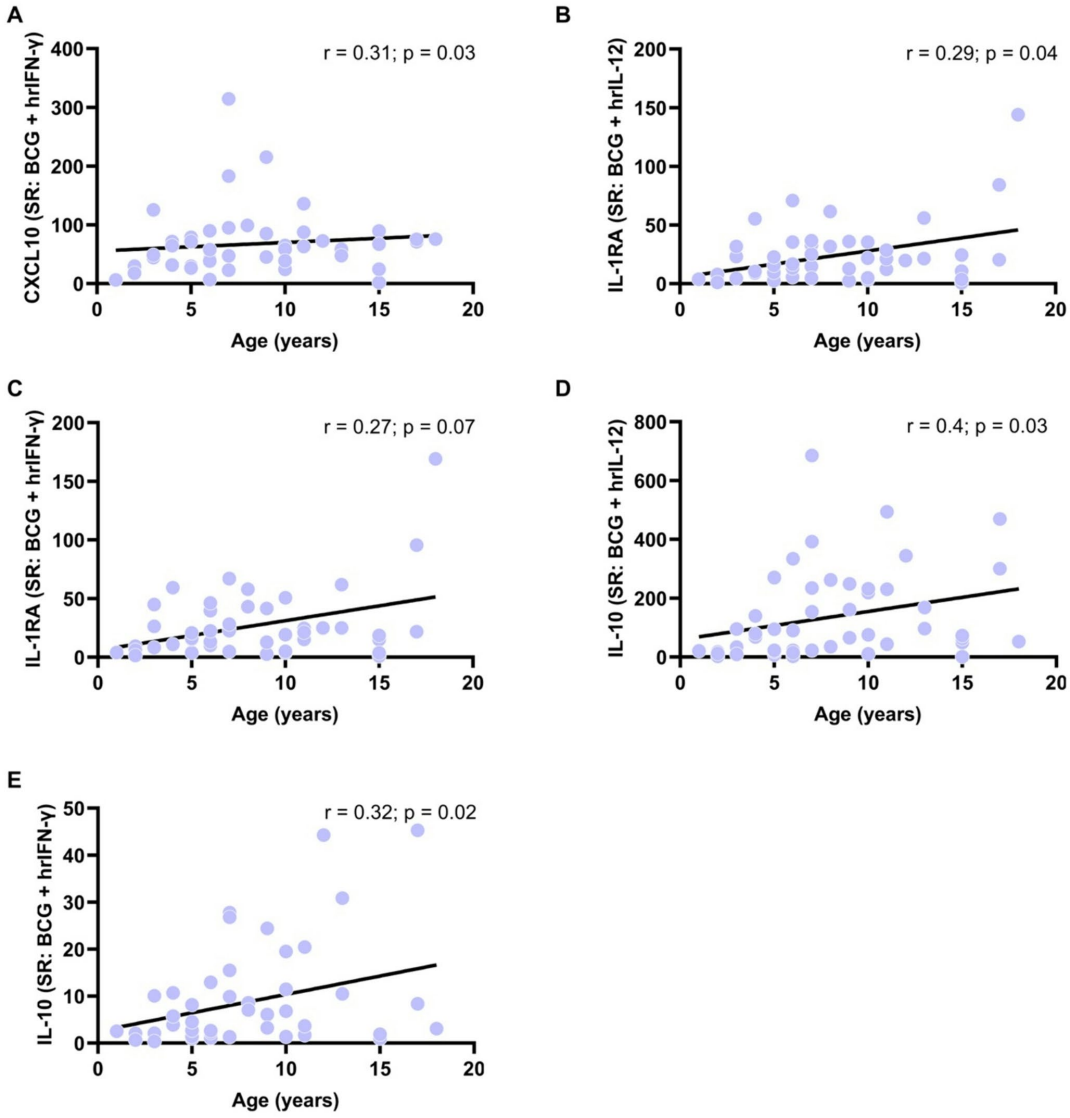
Levels of CXCL10, IL-1RA and IL-10 in a healthy pediatric population (n = 55; ages 1–18 years) correlated with age. A CXCL10 levels (stimulation ratio: SR, calculated as stimulated/basal condition) in response to BCG plus hrIFN-γ. B-E IL-1RA and IL-10 levels (SR) in response to both BCG plus hrIL-12 and BCG plus hrIFN-γ. The levels of all three cytokines increased moderately with age, indicating enhanced IFN-γ downstream signaling (CXCL10) and a parallel rise in inhibitory responses to balance inflammatory activity. Statistical significance p < 0.05; Spearman correlation (r): low association 0.1-0.3; moderate positive association between 0.3-0.5; and strong positive association 0.5-1.

While these cytokine levels remained stable, the capacity to respond to IFN-γ and IL-12 increased with age. Notably, CXCL10 showed a moderate positive correlation with age in response to BCG plus hrIFN-γ (*r* = 0.31 for SR; *p* = 0.03) (Fig. 2A). Interestingly, IL-1RA levels increased with age in response to BCG plus IL-12 (*r* = 0.29 for SR; *p* = 0.04) (Fig. 2B) and showed a trend toward increase with BCG plus hrIFN-γ (*r* = 0.27 for SR; *p* = 0.07; not fully significant) (Fig. 2C). Similarly, IL-10 levels increased moderately with age in response to both BCG plus IL-12 (*r* = 0.4; *p* = 0.03) (Fig. 2D) and BCG plus hrIFN-γ (*r* = 0.32; *p* = 0.02) (Fig. 2E).

### Consistent Expression of IFN-γR1/2 and STAT1 Activity in Monocytes with Age

We assessed IFN-γR1/IFN-γR2 expression on monocytes (CD14+) and found non-significant correlation between IFN-γR expression and age (*p* > 0.05 for both % and MFI) (Figure S8). Descriptive data (Table S5) showed that IFN-γR1 expression ranged from a median of 59.3% to 83.2% across age groups, while IFN-γR2 remained consistently high (median values > 88%). MFI values showed high interindividual variability but no age-related trend. Similarly, pSTAT1/dSTAT1 responses to dose-dependent IFN-γ showed consistent results across ages (*p* > 0.05 for both % and MFI), although a modest positive correlation observed between pSTAT1 activation at 10^2^ IU/mL hrIFN-γ (*r* = 0.29 for %; *p* = 0.05) (Figure S9). As detailed in Table S6, STAT1 phosphorylation in response to IFN-γ (10²–10⁴ IU/mL) showed no age-related differences, with consistent stimulated-to-basal MFI ratios (1.4–2.6) across all groups, indicating stable intracellular signaling. Similarly, STAT1 dephosphorylation dynamics after staurosporine treatment (15–30 min) was stable with age. In summary, IFN-γR1/2 expression and STAT1 activity in monocytes remained stable with age.

### Age-Associated Positive Maturation of T Cell compartment, Including Th Cells

**Fig. 3:**
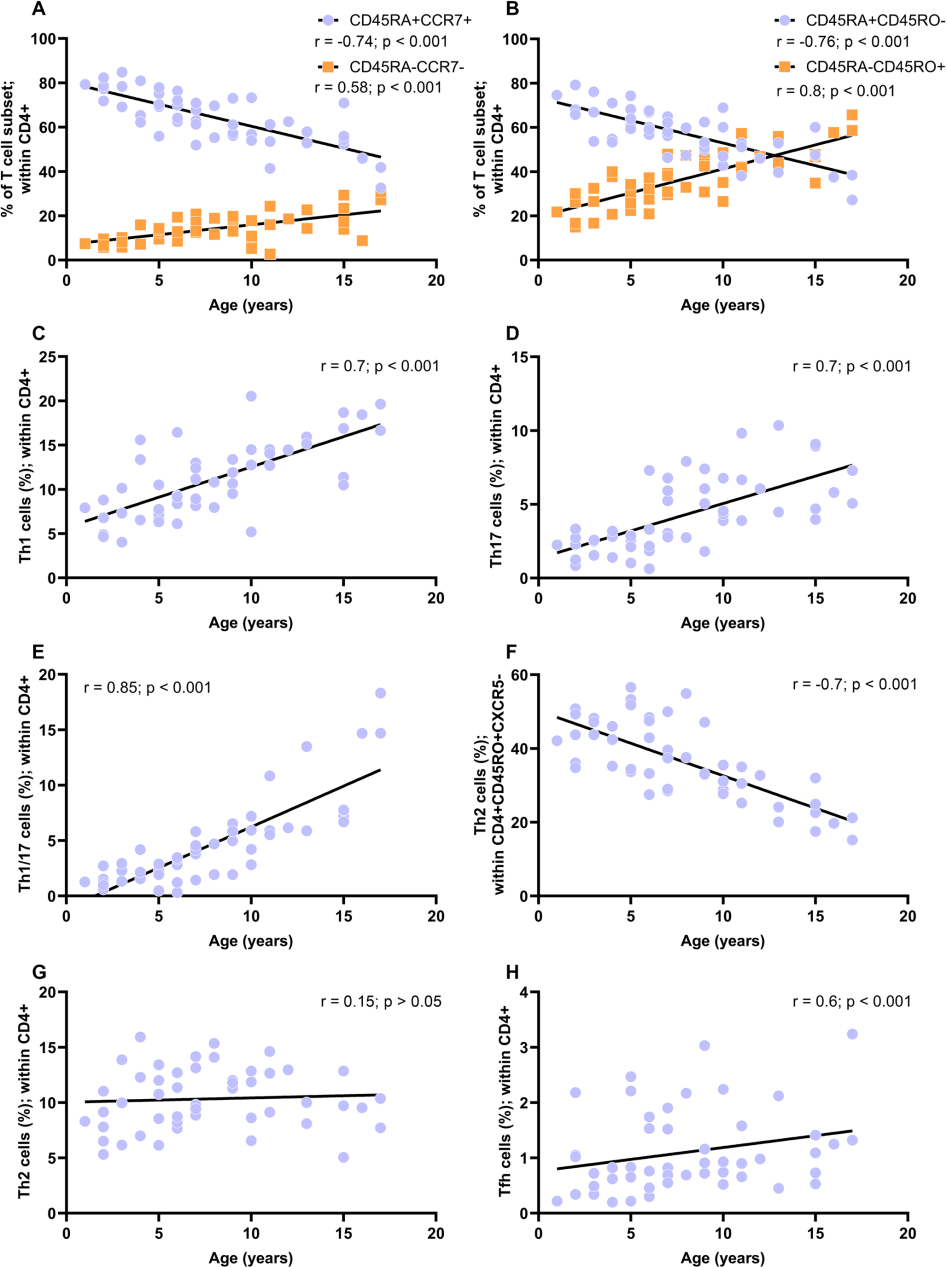
Relative frequency (%) of T cell subsets in a healthy pediatric population (n = 55; ages 1–18 years) correlated with age. A The % of naïve T cells (CD4+CD45RA+CCR7+) decreased with age, along with an increase of effector memory T cells (TEM: CD4+CD45RA-CCR7-) with age. B The % of naïve T cell (CD4+CCD45RA+CD45RO-) decreased with age, along with an increase of late memory T cells (CD4+CD45RA-CD45RO+). C-E Th1 (CD45RA-CD45RO+CXCR5-CXCR3+CCR6+), Th17 (CD45RO+CXCR5-CXCR3-CCR6+) and Th1/17 (CD45RA-CD45RO+CXCR5-CXCR3+CCR6+) cells showed a strong positive correlation with age. F In contrast, Th2 cells (CD45RA-CD45RO+CXCR5-CXCR3-CCR6-) within CD4+CD45RO+CXCR5- cells exhibited a strong negative correlation with age. G However, Th2 cells within CD4+ cells were consistent with age. H T follicular helper (Tfh: CD45RA-CD45RO+CXCR5+) cells also increased with age. Overall, these results suggested a maturation process of T cell compartment. The % of all T cell subsets were calculated within CD4+ cells, except for Th2. Statistical significance p < 0.05; Spearman correlation (r): low association 0.1-0.3; moderate positive association between 0.3-0.5; and strong positive association 0.5-1.

CD3 + CD4 + T cell maturation progressed significantly between ages 1–18 years. This was marked by a pronounced age-related decline in naïve CD4 + T cells [CD4 + CD45RA + CCR7+ (*r* = −0.74; *p* = 9.59E-10) and CD4 + CD45RA + CD45RO- (*r* = −0.76; *p* = 1.18E-10)] and an increase in memory CD4 + T cells [CD4 + CD45RA-CCR7- (*r* = 0.58; *p* = 7.70E-06) and CD4 + CD45RO+ (*r* = 0.80; *p* = 5.73E-12)] (Fig. 3A and B; Table 3). In contrast, maturation within the CD3 + CD8 + T cell compartment was less pronounced (Figure S10). Interestingly, the CD45RA/CCR7 and CD45RA/CD45RO marker combinations showed strong consistency (Figure S11). Specifically, naïve CD4 + T cells identified as CD4 + CD45RA + CCR7 + and CD4 + CD45RA + CD45RO- showed a strong correlation (*r* = 0.93; *p* = 8.57E-21). Similarly, T effector memory (T_EM_) cells identified as CD4 + CD45RA-CCR7- were highly comparable to late memory T cells (CD4 + CD45RA-CD45RO+) with a correlation of *r* = 0.62 (*p* = 4.12E-7).

**Table 3:** T cell subsets frequency (%) from 1–18 years old (yo). All frequencies were calculated within CD4 + cells. Values are presented as median and interquartile range (Q1–Q3)

T cell subsets	1–3 yo (*N* = 9)	> 3–5 yo (*N* = 6)	> 5–7 yo (*N* = 9)	> 7–10 yo (*N* = 14)	> 10–14 yo (*N* = 6)	> 14–18 yo (*N* = 8)	Correlation with age (*r*)	*p*-value
CD3 + CD4 + CD45RA + CCR7 + Naïve Th	78.3 (75.9–80.0)	74.1 (66.5–78.3)	69.3 (62.5–72.0)	62.0 (56.4–70.6)	55.7 (53.0–60.4.0.4)	52.3 (43.8–55.1)	−0.74	9.59E-10
CD3 + CD4 + CD45RA-CCR7- Effector memory Th	7.3 (6.4–8.0.4.0)	11.9 (8.4–15.5)	12.5 (10.6–13.5)	13.4 (11.8–18.6)	17.3 (14.7–21.7)	23.4 (15.6–28.2)	0.58	7.70E-06
CD3 + CD4 + CD45RA + CD45RO- Naïve Th	71.3 (67.1–76.9)	65.4 (57.0–70.0)	60.1 (59.0–66.9.0.9)	54.8 (48.0–61.7.0.7)	48.6 (41.2–52.5)	47.1 (38.0–47.4.0.4)	−0.76	1.18E-10
CD3 + CD4 + CD45RA-CD45RO + Late memory Th	24.1 (16.7–27.6)	26.1 (23.2–34.9)	32.3 (27.5–34.2)	40.9 (31.4–46.9)	45.3 (42.6–53.6)	47.8 (47.2–58.2)	0.8	5.73E-12
CD3 + CD4 + CD45RA-CD45RO + CXCR5-CXCR3 + CCR6- Th1	6.8 (4.8–8.4)	7.2 (6.7–11.9)	9.2 (7.7–10.5)	11.0 (9.1–12.7)	14.5 (14.2–15.0)	16.9 (14.0–18.6.0.6)	0.7	1.44E-08
CD3 + CD4 + CD45RA-CD45RO + CXCR5-CXCR3-CCR6- Th2	8.0 (6.4–9.6)	11.0 (7.7–12.2)	9.4 (8.5–11.4)	11.9 (10.2–13.1)	11.3 (9.3–12.9)	9.7 (8.6–10.0)	0.15	0.31
CD3 + CD4 + CD45RA-CD45RO + CXCR5-CXCR3-CCR6 + Th17	2.3 (1.5–2.6)	2.7 (2.6–2.8)	2.2 (1.8–3.3)	4.8 (3.3–6.6)	6.4 (4.9–9.0.9.0)	5.8 (4.9–8.1)	0.72	5.75E-09
CD3 + CD4 + CD45RA-CD45RO + CXCR5-CXCR3 + CCR6 + Th1/17	1.3 (1.1–1.7)	2.2 (2.0–2.8.0.8)	2.8 (1.2–3.3)	4.6 (3.1–5.8)	6.0 (5.9–9.7)	7.8 (7.5–14.7)	0.85	2.25E-14
CD3 + CD4 + CD45RA-CD45RO + CXCR5 + Tfh	3.5 (2.8–4.7)	3.9 (3.0–4.3.0.3)	5.1 (4.4–7.6)	6.1 (5.0–7.7.0.7)	6.5 (5.5–8.8)	8.2 (6.0–8.6.0.6)	0.59	1.74E-05

Abbreviations: Th: T helper; Tfh: T follicular helper. Spearman correlation (r): low association 0.1–0.3; moderate positive association between 0.3–0.5; and strong positive association 0.5–1.5; Statistical significance *p* < 0.05.

Further evidence of CD3 + CD4 + T cell maturation was shown by strong positive correlations between age and Th cell subset frequencies within CD4 + cells. These included Th1 (CD45RA-CD45RO + CXCR5-CXCR3 + CCR6+; *r* = 0.7; *p* = 1.44E-08), Th17 (CD45RA-CD45RO + CXCR5-CXCR3-CCR6+; *r* = 0.72; *p* = 5.75E-09), and Th1/17 (CD45RA-CD45RO + CXCR5-CXCR3 + CCR6+; *r* = 0.85; *p* = 2.25E-14) (Fig. 3C–E; Table 3). Conversely, the Th2 subset (CD45RA-CD45RO + CXCR5-CXCR3-CCR6-) within CD4 + CD45RO + CXCR5- cells exhibited a strong negative correlation with age (*r* = −0.73; *p* = 0.31) (Fig. 3F; Table 3). On the other hand, Th2 frequency as % of CD4 + cells was maintained with increasing age (Fig. 3G). Additionally, T follicular helper (Tfh; CD45RA-CD45RO + CXCR5+) cells within CD4 + cells showed a strong positive correlation with age (*r* = 0.59; *p* = 1.74E-05) (Fig. 3H). Descriptive data are detailed in Table 3 and Table S7.

Notably, the overall lymphocyte and T cell proliferative capacity in response to mitogens (PHA, ConA and PWD) remained consistent across ages (*p* > 0.05). Further details are provided in Figure S12 and Table S8.

In summary, we observed progressive T cell maturation, including Th1, Th17 and Th1/17 subsets, while T cell proliferative capacity remained stable.

### Critical Developmental Shifts in T Cell Maturation Observed at Ages 6 and 8.6 Years

**Fig. 4:**
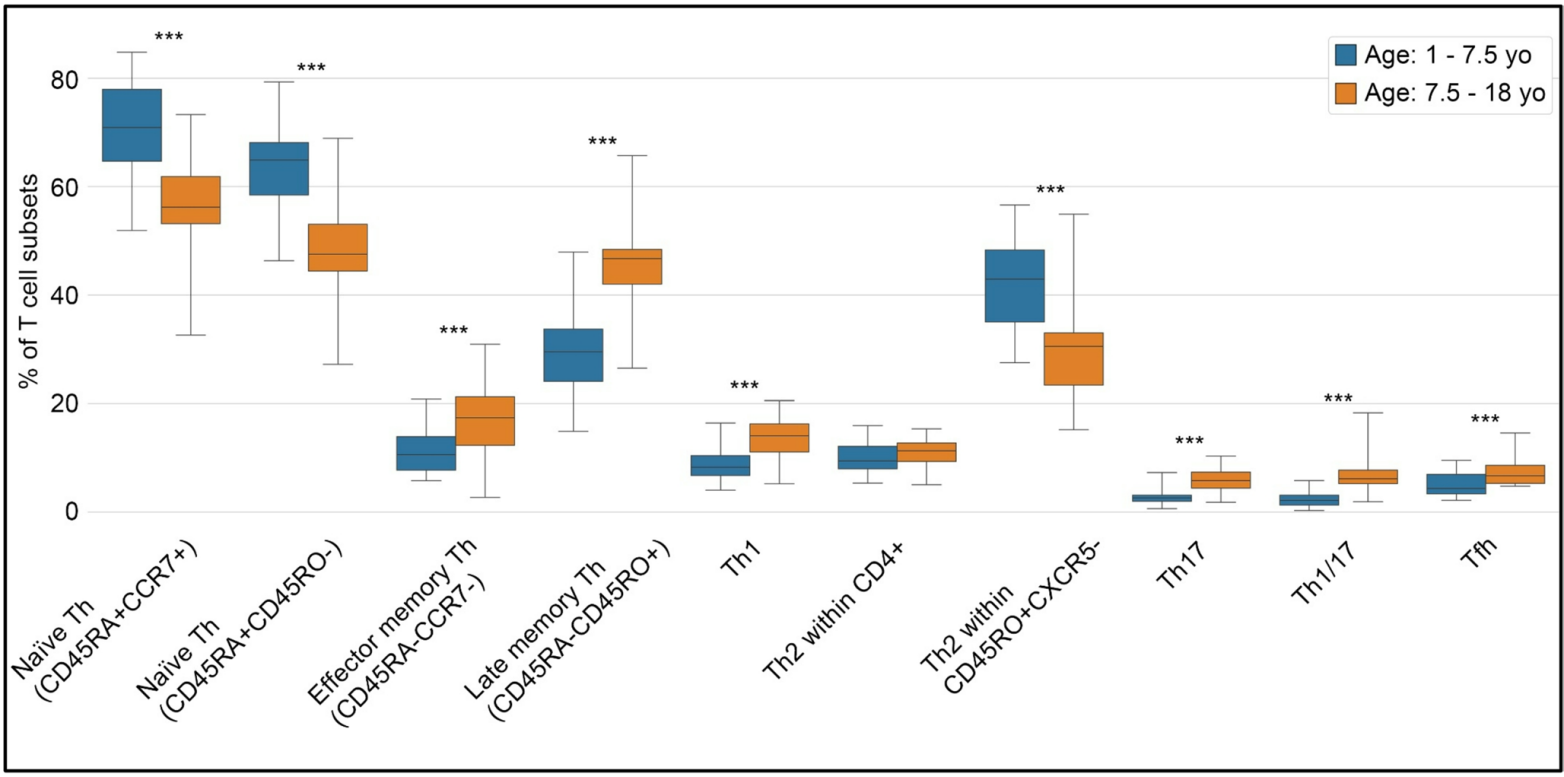
Comparison of T cell subset frequencies (%) between children under 7.5 years old (yo) and above 7.5 yo. The subsets analyzed include: naïve T cells (CD4+CD45RA+CCR7+ and CD4+CD45RA+CD45RO-); memory T cells (CD45RA-CCR7- and CD45RA-CD45RO+); Th1 cells (CD45RA-CD45RO+CXCR5-CXCR3+CCR6+); Th2 cells (CD45RA-CD45RO+CXCR5-CXCR3-CCR6-) within CD4+ and within CD4+CD45RO+CXCR5- cells; Th17 (CD45RO+CXCR5-CXCR3-CCR6+); Th1/17 (CD45RA-CD45RO+CXCR5-CXCR3+CCR6+); and T follicular helper (Tfh: CD45RA-CD45RO+CXCR5+) cells. The findings indicate that 7.5 years marks a pivotal stage in T cell compartment maturation, characterized by a notable decrease in naïve T cells and an increase in memory T cells and Th cell populations. The % of T cell subsets were calculated within CD4+ cells. Statistical significance p < 0.05; *: p < 0.05; **: p < 0.01; ***: p < 0.001.

To investigate age-related shifts in T cell maturation course, we initially grouped children into consecutive 3-year age intervals based on criteria from current literature [59–61] and to ensure balanced group sizes for statistical comparisons. Exploratory analysis (see methods) revealed that the most pronounced changes in T cell subset distribution occurred between approximately 6 and 8.6 years of age. To interrogate this transition more formally, we defined the midpoint of this interval (7.5 years) as a binary cutoff and compared immune parameters in children aged < 7.5 versus ≥ 7.5 years. This analysis confirmed statistically significant differences (*p* < 0.05) in multiple T cell subsets, including naïve and memory CD4⁺ T cells and Th cell populations (Fig. 4), thereby supporting the presence of a developmental inflection in T cell compartment within this age range.

Interestingly, while this age range (6 and 8.6 years) is relatively narrow, we observed that some T cell subsets exhibit earlier maturation time points than others within this period. Specifically, at the age of 6, we noted a marked reduction in naïve T cells (CD4 + CD45RA + CCR7 + and CD4 + CD45RA + CD45RO-) along with a prominent increase in memory T cells (CD4 + CD45RA-CCR7+, CD4 + CD45RA-CCR7- and CD4 + CD45RA-CD45RO+) as shown in Fig. 3. In parallel, we observed an abrupt increase of Th17 and Tfh cells at age 6. In contrast, Th1 and Th1/17 cells exhibited a marked increase at age 8.6 (Fig. 3).

### IFN-γ Levels Showed no Correlation with Th1 and Th1/17 Cells

As Th1, Th17, and Th1/17 cells are key IFN-γ producers [47, 62], we analyzed their correlation with IFN-γ levels in plasma and after BCG plus hrIL-12 stimulation. Basal IFN-γ levels in plasma (ELISA) showed no correlation with Th1 (*r* = 0.1; *p* = 0.53) or Th17 cells (*r* = 0.19; *p* = 0.2), and similar results were observed for IFN-γ levels after BCG plus IL-12 stimulation (ELISA and Luminex; *p* >0.05) (Figure S6 and Figure S7). Similarly, we observed no association between CXCL10 with Th1 (*r* = 0.06; *p* = 0.7) or Th1/17 (*r* = 0.29; *p* = 0.05), only a moderate correlation with Th17 (*r* = 0.34; *p* = 0.02) (Figure S7).

## Discussion

This study provides the first integrated analysis of type II IFN immunity across childhood (1–18 years), with direct implications for diagnosing MSMD and other IEIs involving IFN-γ pathways. We show that the core components of the IL-12/IFN-γ axis (IFN-γ, IL-12, TNF, IFN-γR1/2 expression, and STAT1 signaling) remain stable throughout pediatric development, indicating that results from children can be reliably interpreted using adult reference values. In contrast, downstream signaling and effector responses evolve with age: CXCL10 production progressively increases, while sequential shifts in Th subsets (with critical transitions at 6–8.6 years) mark the maturation of adaptive immunity. Importantly, the lack of strong correlation between Th1 frequencies and IFN-γ/CXCL10 levels suggests that regulatory cytokines such as IL-1RA and IL-10 play a modulatory role in maintaining immune balance. Together, these findings delineate which aspects of the pathway are developmentally stable versus age-dependent, providing clinicians with a clearer framework to distinguish physiologic maturation from pathological dysfunction when evaluating children with suspected MSMD.

Our findings support and extend current MSMD diagnostic practices. The standard workup includes IFN-γ, IL-12, and TNF levels, IFN-γR1/2, IL-12 receptor (IL-12R)-β1, and STAT1 activity [6, 13]. Due to the lack of pediatric reference values, we defined preliminary thresholds in healthy children, except for IL-12Rβ1, which is binary [6, 63]. Basal IFN-γ levels remained low (< 100 pg/mL) and stable across ages, consistent with uninfected individuals [6, 55]. Similarly, cytokine responses to BCG plus IL-12 or IFN-γ, IFN-γR1/2 expression, and STAT1 activity in monocytes showed no age-related variation. Although STAT1 activation in CD4 + T cells increases during infancy [64], we confirmed stable monocyte activation beyond one year of age. These results suggest consistent IL-12/IFN-γ axis function across childhood, supporting the notion that impaired BCG responses in children (in vitro) are due to intrinsic immune defects rather than developmental immaturity [65].

From a biological perspective, additional cytokines and T cell populations are needed to deepen our understanding of Type II IFN immunity. While our focus was on BCG-induced non-specific responses, it is known that BCG vaccination also affects adaptive immunity by boosting Th1 responses and reducing Th2 activity, highlighting its broader role in shaping immune development [66, 67]. This shift toward Th1 dominance parallels our observations of increasing CXCL10 production and Th1 cell frequency with age, forming a positive feedback loop that reinforces Th1 polarization [48, 51]. The increase in CXCL10, despite stable IFN-γ levels, indicates an age-related enhancement of downstream IFN-γ signaling, highlighting CXCL10’s potential as a tuberculosis marker in children [52, 68]. Thus, while upstream components are stable, the amplification of downstream responses reflects the maturation of adaptive immunity.

Anti-inflammatory cytokines increased with age and may regulate IFN-γ responses. The age-related increase in CXCL10 may trigger negative feedback mechanisms to prevent detrimental inflammatory responses [69, 70], thereby explaining the stability of IFN-γ levels in this study. Specifically, there was a modest age-related increase in IL-1RA production upon BCG plus IFN-γ or IL-12, indicating a consistent capacity to regulate IFN-γ-induced inflammation [49]. Similarly, IL-10, a key anti-inflammatory cytokine induced by IFN-γ signaling [70], also increased with age, aligning with the maturation of regulatory T cells [5]. These findings point to a coordinated maturation of immune regulation during childhood. The age-related increase in IL-10 and IL-1RA likely helps modulate IFN-γ responses and maintain immune balance [71]. Although no direct links were found between these cytokines and Th1 cells, their parallel rise with Th1 maturation suggests a synchronized development of immune control and stable IL-12/IFN-γ axis function.

T cell maturation is a key feature of adaptive immune development. Our study underscores the progressive shift from naïve to memory CD4 + T cells, alongside increasing frequencies of Th1, Th17, Th1/17, and Tfh cells. Early-life bias toward Th2 responses is gradually replaced by Th1 and Th17 expansion, aligning with prior studies [4]. The results revealed two key developmental shifts in the T cell compartment: at age 6, there was a marked decline in naïve T cells and an increase in memory T cells, Th17, and Tfh cells; at age 8.6, Th1 and Th1/17 cells rose significantly. These findings align with previous reports showing a decline in naïve T cells (CD45RA + CCR7+) and a rise in effector memory T cells (TEM: CD45RA − CCR7−) from age 7 [60]. These observations support the identification of 6–8.6 years as a key period in T cell maturation, marked by sequential changes in memory CD4 + T cells and effector Th subsets, reflecting a coordinated advancement of the adaptive immune landscape during mid-childhood [72]. Importantly, lymphocyte proliferative capacity remained stable [20, 73], suggesting the observed changes reflect subset distribution rather than global functional decline.

This study offers technical insights into T cell phenotyping, showing a strong correlation between CD45RA/CCR7 and CD45RA/CD45RO in naïve/memory classification. Although subset frequencies varied in scale, both markers showed similar developmental trajectories for naïve/memory subsets. Current recommendations support using CD45RA/CCR7 [17, 74], but our findings suggested that including CD45RO enhances identification of transitional subsets, which can better capture T cell maturation process [75, 76]. Until standardized guidelines are established, clinical settings should customize age-stratified reference values according to the markers used.

Despite the valuable insights provided, this study has several limitations. The sample size was small, and all participants were of White-European origin, without sex-based analysis. Since immune responses can vary by ethnicity and sex due to genetic, environmental, and socioeconomic factors [59, 60, 77–79], broader studies are needed to define reference values in more diverse populations. However, we expect the results to be comparable. Additionally, as this was a cross-sectional study, longitudinal follow-up would offer deeper insights into immune development over time [80]. Future research should also explore downstream effectors of the IL-12/IFN-γ pathway, such as downstream effector molecules, to capture the full scope of immune dynamics in childhood [81, 82]. In addition, studying NK cells, which are also key producers of IFN-γ [44], may offer deeper mechanistic insights of IFN-γ signaling.

## Conclusions

Taken together, we demonstrate that the IL-12/IFN-γ axis remains stable throughout childhood, supporting the use of adult reference values when evaluating pediatric patients with suspected MSMD. In contrast, downstream signaling (CXCL10) and Th cell maturation show age-dependent shifts, particularly between 6 and 8.6 years, highlighting a critical developmental window. These findings provide clinicians with age-specific reference points to distinguish normal immune maturation from pathological dysfunction in children at risk of intramacrophagic infections. Future research should focus on validating these findings in larger and more diverse cohorts, and on developing age-adapted reference ranges to improve diagnostic accuracy in pediatric immunology.

## Supplementary Information

Below is the link to the electronic supplementary material.

Supplementary Material 1

## Data Availability

No datasets were generated or analysed during the current study.

## Declarations

### Submission Declaration and Verification

This manuscript has not been published previously, and it is not under consideration for publication elsewhere. It will not be published elsewhere in the same form, in English or in any other language, including electronically.

All figures are original. The figures created with *BioRender.com* are licensed for submission and publication.
