# Supplementary material for: Age-Related Patterns of Type II Interferon Immunity: Implications for Intramacrophagic Infections and MSMD Diagnosis During Childhood
Source: J Clin Immunol. 2025 Dec 26;46(1):13. doi: 10.1007/s10875-025-01955-2 (PMC12831799; doi:10.1007/s10875-025-01955-2)
Supplement: Supplementary file 1 — Supplementary Material 1 [file 10875_2025_1955_MOESM1_ESM.pdf]

Age-related patterns of type II interferon immunity: implications for intramacrophagic infections and MSMD diagnosis during childhood.

Yiyi Luo[1,2,3], Guillermo Argüello[4,5], Daniel Acevedo[1,2,3], Cristina Jou[6], Anna Codina[6], Jesús Márquez[6], Alexandru Vlagea[3,7], Sara Peiró[7], Víctor Bolaño[7], Aina Freixedas[1,2,3], Angela Deyà-Martínez[1,2,3,8], Ana García-García[1,2,3], Celia Martí-Castellote[1,2,3], Manel Juan[3,7,8], Ana Esteve-Solé[1,2,3], Laia Alsina[1,2,3,8].

Aff1. Pediatric Allergy and Clinical Immunology Department, Clinical Immunology and Primary Immunodeficiencies Unit, Hospital Sant Joan de Déu; Barcelona, Spain, EU.
Aff2. Study Group for Immune Dysfunction Diseases in Children, Institut de Recerca Sant Joan de 30 Déu (IRSJD); Barcelona, Spain, EU.
Aff3. Clinical Immunology Unit, Hospital Sant Joan de Déu-Hospital Clínic; Barcelona, Spain, EU.
Aff4. MedSavana SL, Madrid, Spain.
Aff5. Faculty of Computer Science, Multimedia and Telecommunications, Universitat Oberta de Catalunya, Barcelona, Spain.
Aff6. Biobank and Pathology department. Institut de Recerca Sant Joan de Déu, Hospital Sant Joan de Déu, CIBERER ISCIII. Barcelona 08950, Spain.
Aff7. Immunology Department, Biomedic Diagnostic Center (CDB), Hospital Clinic of Barcelona, Barcelona, Spain.
Aff8. Department of Surgery and Surgical Specializations, Faculty of Medicine and Health Sciences, University of Barcelona; Barcelona, Spain, EU.

***Ana Esteve-Solé and Laia Alsina should be considered joint senior authors.*
The authors of this publication are members of the European Reference Network ERN-RITA.

Corresponding authors (both LA and YL)

L.Alsina
Email: laia.alsina@sjd.es

Y.Luo
Email: yiyi.luo@sjd.es

Full postal address for both:

Fundació Sant Joan de Déu
c/ Santa Rosa 39-57, 3ª Planta
Esplugues de Llobregat, Barcelona, Spain.

TABLE OF CONTENT

EXTENDED METHODS 4

IL-12/IFN-γ axis activity in response to mycobacterial stimulus 5

- Whole blood culture 5
- Cytokine profile in response to mycobacterial challenge (supernatants from the culture) 5
- IFN-γ levels in plasma and supernatants from the culture 5

IFN-γ receptor (IFN-γR) 1 and 2 expression. 6

Signal transducer and activator of transcription (STAT) 1 phosphorylation. 6

Multiparameter flow cytometric identification of T cell subsets. 6

T cell proliferation capacity. 6

Sample acquisition. 7

Statistical analysis. 7

SUPPLEMENTARY TABLES 8

Table S1 List of monoclonal antibodies (mAbs). 9

Table S2 Reagents for immunoassays. 10

Table S3 T cell subset definition. 11

Table S4A. Cytokine levels - IFN-γ, IL-12p70, TNF, CXCL10, IL-RA, IL-10, IL-1β and IL-6 - in basal condition from 1 - 18 years old (yo). 12

Table S4B. Cytokine levels - IFN-γ, IL-12p70, TNF CXCL10, IL-RA, IL-10, IL-1β and IL-6 - in response to phorbol myristate acetate (PMA) plus Ionomycin and tumor necrosis factor (TNF) from 1 - 18 years old (yo). 13

Table S5 IFN-γ receptors (IFN-γR)-1/2 expression in monocytes (CD14+) from 1 - 18 years old (yo). 15

Table S6 Signal transducer and activator of transcription (STAT)-1 phosphorylation and dephosphorylation capacity in a IFN dose dependent manner in monocytes (CD14+) from 1 - 18 years old (yo). 16

Table S7 T cell subsets frequency (%) from 1 - 18 years old (yo). 17

Table S8 T cell proliferative capacity from 1 - 18 years old (yo). 19

SUPPLEMENTARY FIGURES 20

Figure S1 Graphical summary of the methods employed for immune assays 21

Figure S2 IFN-γ receptor (IFN-γR) 1 and 2 expression gating strategy. 22

Figure S3 Signal transducer and activator of transcription (STAT1) 1 phosphorylation and dephosphorylation capacity gating strategy. 23

Figure S4 T cell compartment immunophenotyping gating strategy 24

Figure S5 Proliferation assay gating strategy. 27

Figure S6 Correlation of IFN-γ levels with age and T helper (Th) cells subsets. 28

Figure S7 Correlation of cytokine levels - IFN- γ, IL-10, IL-12p70, IL-1β, IL-1RA, IL-6, CXCL10, TNF - with age and T helper (Th) cells subsets. 29
Figure S8 Correlation of IFN-γ receptors (IFN-γR)-1/2 with age and T helper (Th) cells subsets 31
Figure S9 Correlation of Signal transducer and activator of transcription (STAT)-1 with age and T helper (Th) cells subsets. 32
Figure S10 Correlation of T cell subsets with age 33
Figure S11 Correlation of T cell subsets defined with CD45RA/CCR7 vs CD45RA/CD45RO 34
Figure S12 Correlation of T cell proliferative capacity with age and T helper (Th) cells. 35
SUPPLEMENTARY REFERENCES 36

EXTENDED METHODS

The graphical summary of the methods employed for immune assays is presented in Figure S1. Details of all reagents and monoclonal antibodies (mAbs) utilized in the study are provided in Tables S1-2, respectively.

IL-12/IFN-γ axis activity in response to mycobacterial stimulus.

- *Whole blood culture*

Heparinized whole blood culture was performed within less than 24h after sample collection to analyze the cytokine profile. Heparinized whole blood was diluted 1:2 in complete medium [RPMI (Gibco, Grand Island, NY, USA) supplemented with 10% heat-inactivated fetal calf serum (FCS; Sigma-Aldrich, St. Louis, MO, USA), 1 μg/ml penicillin, and 1μg/ml streptomycin (Invitrogen, Grand Island, NY, USA)] and incubated with different stimulation conditions at 37°C in a 5% CO_2 humidified incubator for 48h. There were 6 stimulation conditions: 1) medium alone with heparinized whole blood (baseline control), 2) live BCG (M. bovis BCG, Pasteur substrain) at a multiplicity of infection of 20 BCG per leukocyte, 3) BCG plus human recombinant (hr) IL-12p70 (20 ng/ml, Miltenyi Biotec, Germany), 4) BCG plus hrIFN-γ (5,000 IU/ml; Imukin, Boehringer Ingelheim, Germany), 5) phorbol myristate acetate (PMA, 50ng/mL, Sigma-Aldrich, St. Louis, USA) plus ionomycin (1ug/mL, Sigma-Aldrich, St. Louis, USA), and 6) TNF (10μg/mL, Milteny Biotec, Bergisch Gladbach, Germany).

- *Cytokine profile in response to mycobacterial challenge (supernatants from the culture)*

Cytokine production—specifically IFN-γ, IL-12p70, TNF, IL-1 receptor antagonist (IL-1RA), IL-1β, interferon-inducible protein (IP)-10, IL-6, and IL-10—was assessed after determining the culture supernatants at the 18h culture point (1). This assessment was performed using the Luminex platform (Millipore, Billerica, MA, USA), following the manufacturer's instructions (2,3). Briefly, supernatants from the culture were incubated for 2h with corresponding anti-cytokine magnetic beads, and then washed with 1X washing buffer and stained with detection antibodies (provided) for 1h. Strepatividin-PE was then added for 30 more minutes. During all incubation steps, the plate was agitated at 650 rpm. After washing, plate was agitated for 15 min at 650 rpm and read in the xMAP Luminex reader (Waltham, MA, USA). The normalization of the results must consider the heparinized whole blood dilution 1:2 in complete medium and the cells (lymphocyte and monocytes) that release the cytokines.

- *IFN-γ levels in plasma and supernatants from the culture*

IFN-γ level in plasma (baseline condition) and culture supernatants, at 48h culture point, was analyzed by enzyme-linked immunosorbent assay (ELISA, Invitrogen, Grand Island, New York, NY, USA) (1) following the manufacture instructions. Briefly, a 96 well plate was coated with IFN-γ capture antibody overnight at 4ºC. The plate was washed (x3, following the manufacturer's instructions) with wash buffer [phosphate buffered saline (PBS) 1X plus 0.05 of Tween (Millipore, Billerica, MA, USA)], the plate was cultured with 200μL of assay buffer for 1h to block non-specific interactions and washed again. 100μL of standards and samples (from culture and plasma) were added to the plate and incubated for 2h. Subsequently, the plate was washed (x4) and incubated with detection antibody for 1h. After washing (x4), horseradish peroxidase-conjugated

streptavidin enzyme was added and incubated for 30min. Following the plate washing (x6), 3,3',5,5'-tetramethylbenzidine was added and incubated for 15min at dark. Finally, the enzymatic reaction was stopped by H_2SO_4 and read in BioTek Epoch ELISA Reader in 450nm wavelengths. During all incubation steps, the plate was agitated at 400 rpm.

IFN-γ receptor (IFN-γR) 1 and 2 expression.

IFN-γR1/IFN-γR2 expression was evaluated by flow cytometry including an unstained control for gating purposes. 50μL of heparinized heparinized whole blood of both conditions was incubated for 15 min with 5μL inactivated goat serum to avoid the non-specific binding of the monoclonal antibodies (mAbs) to the sample. Following, the study condition cells were stained with mAbs (IFN-γR-1-PE and IFN-γR-2-APC) for 30 min at RT. To lyse erythrocytes and fix cells, the cells were incubated with 2 mL of lysing solution-1X (BD Bioscience) for 15 min at RT. Cells were then washed two times with PBS-1X at 1500rpm for 5 min.

Signal transducer and activator of transcription (STAT) 1 phosphorylation.

STAT1 phosphorylation and dephosphorylation capacity was evaluated by flow cytometry (Figure S1). Specifically, 50μL of heparinized whole blood was stimulated for 15 min with: 1) 10^2 IU/mL hrIFN-γ; 2) 10^4 IU/mL hrIFN-γ; 3) 10^4 IU/mL hrIFN-γ followed by washing and incubation with staurosporine (0.5μM, Sigma-Aldrich, St. Louis, MO, USA) for 15 min; 4) 10^4 IU/mL hrIFN-γ followed by washing and incubation with staurosporine for 30 min; 5) 10^3 IU/mL IFN-α (Pegasys, Roche, Paris, France); the blood was washed twice with RPMI. Then, we lysed the erythrocytes and fixed/permed the cells. To lyse erythrocytes and fix cells, the cells were incubated with 500μL of Lyse/Fix solution-1X (BD Bioscience) for 15 min at 37ºC and washed two times with RPMI. Then, the cells were permeabilized with 500μL of Perm Buffer III (BD Bioscience) for 30 min at 4ºC; the buffer is added dropwise while slowly vortexing. Finally, the cells were stained with anti-phosphorylated STAT1-PE and CD14-APC for 30 minutes at RT and 30 min at 4ºC and washed with PBS-1X.

Multiparameter flow cytometric identification of T cell subsets.

We studied the T cell compartment subsets by multiparameter immunophenotyping (4,5). For cell staining, 50μL of heparinized whole blood was incubated for 15 min at RT with the appropriate mAbs (Table S1). To lyse erythrocytes and fix cells, stained cells were incubated with 2 mL of lysing solution-1X for 15 min at RT. Finally, cells were washed two times with PBS-1X.

T cell proliferation capacity.

Peripheral blood mononuclear cells (PBMCs) were isolated by Ficoll-Hipaque (Sigma-Aldrich, St. Louis, MO, USA) density gradient centrifugation of heparinized whole blood (6). Cells were subsequently washed three times with PBS-1X and cultured with RPMI. Viable cells were counted using a hemocytometer in an inverted microscope. Then, $1.5 \cdot 10^6$ PBMC/mL were stained with carboxifluorescein diacetate succinimidyl ester (CFSE, Invitrogen, Grand Island, NY, USA) (3,7) and stimulated with mitogen for 7 days at 37ºC (5% CO_2). Specifically, $1.5 \cdot 10^6$ peripheral blood mononuclear cells (PBMC)/mL were labeled with 5μM with CFSE (Invitrogen, Grand Island, NY, USA)

(3,7) for 10 min at 37ºC; then 5mL of cold PBS supplemented with 10% FCS was added for 5 min at 4ºC and washed twice with PBS + 2% FCS before stimulation. CFSE-stained PBMCs were stimulated with phytohemaglutinin A (5µg/mL; PHA, Sigma, St. Louis, MO, USA), PHA plus IL-2 (PHA: 5µg/mL; IL-2: 1,250 IU/mL, Hospital Clínic pharmacy), pokeweed mitogen (2 µg/mL; PWM; Sigma, St. Louis, MO, USA) and Concanavalin A (2 µg/mL; ConA, Sigma, St. Louis, MO, USA) or medium only in a 96-well plate for 7 days at 37ºC (5% CO_2). After 7 days, cells were stained with mAbs (CD4-FITC; CD19-Pe-Cy7; CD3-APC-H7) for 15min at RT and washed with 2mL of PBS-1X. We analyzed division index (DI) and proliferation index (PI) calculated with FlowJo v.10.

Sample acquisition.

All samples studied by flow cytometry were acquired using FACSCanto-II (BD Bioscience) cytometer. A minimum of 20,000 events were acquired for the different population studied: T cells for T cell subsets and proliferation assay, and monocytes for STAT1 detection and IFN-γR-1/IFN-γR-2. Flow cytometry data were analyzed by Flowjo v.10. The Luminex results were read using xMAP Luminex reader (Waltham, MA, USA). ELISA results were read using BioTek Epoch ELISA Reader at 450nm wave lengths.

Statistical analysis.

Statistical analyses included summarizing categorical variables as frequency (n) and percentage (%) and reporting non-normally distributed continuous variables as median (Mdn) and interquartile range (IQR). Since variables did not follow a normal distribution, group comparisons were performed using the Kruskal-Wallis test, with significance set at $p < 0.05$, and correlations analyzed using Spearman's rank coefficient, presented in a full correlation matrix. Spearman test: the perfect negative correlation is referred as -1 and the perfect positive correlation as +1. Low positive association 0.1-0.3; moderate positive association between 0.3-0.5; and strong positive association 0.5-1 (8). Negative correlations follow the same criteria. All analyses were conducted in Python 3.12 (Python Foundation, USA). We used Python 3.12 and Prism 7.04 software (GraphPad, La Joll a, CA, USA) for the graphical representation.

To explore potential non-linear age-related patterns in T cell maturation, we performed exploratory analyses by stratifying participants into consecutive 3-year age intervals, based on prior literature and cohort distribution. Between-group differences in immune parameters across these age brackets were assessed using the Kruskal–Wallis test for continuous variables. When patterns suggested potential inflection points, a data-driven binary cutoff (7.5 years) was defined at the midpoint of the interval showing the greatest variability. Immune parameters were then formally compared across this cutoff using the Mann–Whitney U test to confirm statistical significance of the observed transitions.

SUPPLEMENTARY TABLES

Table S1 List of monoclonal antibodies (mAbs). Multiparameter flow cytometry panels to define the T cells, IFN-γ receptors (IFN-γR)1/2, signal transducer and activator of transcription (STAT)-1 and T cell proliferation assay. Final volume for the test was 50 µL; for T cell proliferative assay was 100 µL.

Panel	Surface marker	Fluorocrom	Company	Catalog Number	Volume/ Test (µL)
T cells	TCR αβ	FITC	BD	333140	2
	TCR γδ	PE	BD	333141	2
	CD8	PerCP-Cy5.5	BD	565310	2
	CD45RA	Pe-Cy7	BD	337186	2
	CD45RO	APC	BD	340438	2
	CD4	APC	BD	345771	2
	CD3	APC-H7	BD	641415	2
	HLA-DR	V450	BD	655874	2
	CCR7	BV510	BD	566760	2
T helper cells	CD4	FITC	BD	345768	2
	CXCR3	PE	BD	550633	10
	CD8	PerCP-Cy5.5	BD	565310	2
	CCR6	Pe-Cy7	BD	560620	2
	CD45RO	APC	BD	340438	2
	CD45RA	APC-H7	BD	561212	2
	CD3	V450	BD	560365	2
	CXCR5	BV510	BD	563105	2
IFNγ receptors	IFNγR1	PE	BD	558937	2
	IFNγR2	APC	R&D systems	FAB773A	10
STAT1 phosphorylation dephosphorilation	STAT 1	PE	BD	612564	6
	CD14	APC	BD	555399	6
T cell proliferation assay	CD4	FITC	BD	345768	2
	CD19	Pe-Cy7	BD	557835	2
	CD3	APC-H7	BD	641415	2

Table S2 Reagents for immunoassays.

Assay	Reagent name	Company	Catalog number	Test*
Flow cytometry lysing	Lyse solution	BD	349202	2mL
IL-12/IFN-γ axis culture	BCG	-	-	20 BCG/leukocyte
	hrL-12p70	Miltenyi Biotec	130-096-798	20 ng/ml
	hrIFN-γ Imukin	Boehringer Ingelheim	-	5,000 IU/ml
	TNF	Miltenyi Biotec	130-094-014	10 µg/mL
	PMA	Sigma Aldrich	P8139-1MG	50 ng/mL
	Ionomycin	Sigma Aldrich	i0634-1MG	1 ug/mL
T cell proliferation	CFSE	Invitrogen	C34554	5µM
	IL-2	Hospital Clínic pharmacy	-	1,250 IU/mL
	PHA	Sigma Aldrich	L1668-5MG	5µg/mL
	ConA	Sigma Aldrich	C5275-5MG	2 µg/mL
	PWM	Sigma Aldrich	L8777-10MG	2 µg/mL
Luminex:	Human Cytokine Magnetic Bead Panel	Millipore	HCYTOMAG-60K	MI
IFN-γ levels	IFN-γ Human Uncoated ELISA Kit	Invitrogen	88-7316-88	MI
	Tween	Millipore	9005-64-5	0.05%
STAT1 activity	Lyse/Fix Buffer 5x	BD	558049	500µL
	Perm Buffer III	BD	558050	500µL
	Human recombinant IFN-γ, Imukin	Boehringer Ingelheim	-	10^2 and 10^4 IU/mL
	Pegasys (peginterferon α)	Pegasys, Roche	-	10^3 IU/mL

Test: volume or concentration/test. Abbreviations: BCG: bacille calmette-guérin; BD: Becton Dickinson (company); CFSE: carboxifluorescein diacetate succinimidyl ester; ConA: concavalin A; ELISA: enzyme-linked immunosorbent assay; hrIFN-γ: human recombinant IFN-γ; hrIL-12p70: human recombinant IL-12p70; MI: manufacturer's instructions; PHA: phytohemaglutinin A; PWM: pokeweed mitogen; PMA: phorbol myristate acetate; Signal transducer and activator of transcription (STAT).*

Table S3 T cell subset definition.

Lymphocyte subset name	Cluster of differentiation (CD)
**T cell**	CD3+
TCR αβ	CD3+TCR αβ
TCR γδ	CD3+TCR γδ
**CD3+CD4+**	CD3+CD4+
Naïve T cells	CD3+CD45RA+
Naïve Th	CD3+CD4+CD45RA+CCR7+
Naïve Th	CD3+CD4+CD45RA+CD45RO-
Central memory Th	CD3+CD4+CD45RA-CCR7+
Effector memory Th	CD3+CD4+CD45RA-CCR7-
Effector memory RA Th	CD3+CD4+CD45RA+CCR7-
Early memory	CD3+CD45RA+CD45RO+
Late memory	CD3+CD45RA-CD45RO+
Late memory Th	CD3+CD4+CD45RA-CD45RO+
Activated Th	CD3+CD4+HLA-DR+
Terminally differentiated Th	CD3+CD4+CD57+
Activated Th	CD3+CD4+CD69+
Th1	CD3+CD4+CD45RA-CD45RO+CXCR5-CXCR3+CCR6-
Th2	CD3+CD4+CD45RA-CD45RO+CXCR5-CXCR3-CCR6-
Th17	CD3+CD4+CD45RA-CD45RO+CXCR5-CXCR3-CCR6+
Th* or Th1/17	CD3+CD4+CD45RA-CD45RO+CXCR5-CXCR3+CCR6+
Tfh	CD3+CD4+CD45RA-CD45RO+CXCR5+
**CD3+CD8+**	CD3+CD8+
Naïve Tc	CD3+CD8+CD45RA+CCR7+
Naïve Tc	CD3+CD8+CD45RA+CD45RO-
Central memory Tc	CD3+CD8+CD45RA-CCR7+
Effector memory Tc	CD3+CD8+CD45RA-CCR7-
Effector memory RA Tc	CD3+CD8+CD45RA+CCR7-
Activated Tc	CD3+CD8+HLA-DR+
Late memory Tc	CD3+CD8+CD45RA-CD45RO+
Terminally differentiated Tc	CD3+CD8+CD57+
Activated Tc	CD3+CD8+CD69+
Double negative T	CD3+CD4-CD8-

Abbreviations: CCR: chemokine receptor; CXCR: C-X-C chemokine receptor; TCR: T cell receptor; Tc: T cytotoxic cells; Tfh: T follicular helper cells; Th: T helper cells.

Table S4A. Cytokine levels - IFN-γ, IL-12p70, TNF, CXCL10, IL-RA, IL-10, IL-1β and IL-6 - in basal condition from 1 - 18 years old (yo). Values are presented as median and interquartile range (Q1–Q3). Values were normalized

Cytokine	1-3 yo (N = 9)	>3-5yo (N = 6)	>5-7yo (N = 9)	>7-10yo (N = 14)	>10-14yo (N = 6)	>14-18yo (N = 8)
IFN-γ	3.85 (1-7.87)	1 (0.89-8.4)	4.69 (0.94-17.65)	1.04 (0.26-10.51)	1.7 (1-14.55)	1.19 (0.88-27.55)
IL-10	9.13 (0.36-40.63)	0.84 (0.53-10.37)	1.37 (0.42-17.87)	1.06 (0.07-14.49)	1.72 (0.73-17.37)	1.98 (0.44-22.93)
IL-12	3.85 (0.11-9.97)	0.54 (0.07-8.4)	2.13 (0.48-11.11)	1 (0-7.43)	0.98 (0.1-12.21)	0.83 (0.11-17.83)
IL-1RA	8.33 (2.37-44.91)	12.46 (4.1-65.19)	14.32 (8.05-26.44)	10.13 (3.71-60.65)	14.07 (11.12-21.81)	24.5 (1-96.82)
IL-1B	6.36 (0.04-104.99)	1 (0.45-7.41)	1 (0.37-12.77)	1 (0.14-35.83)	0.85 (0.26-15.02)	1 (0.04-2077.71)
IL-6	14.76 (0.09-5834.12)	0.61 (0.23-11.85)	1 (0.11-22.86)	1.07 (0.17-182.48)	1 (0.18-21.13)	1.03 (0.16-13726.75)
CXCL10	350 (35.79-1613.59)	103.55 (50.95-367.41)	224.02 (53.45-2240.52)	111.27 (16.59-555.24)	170.42 (103.5-255.4)	191.71 (123.7-451.32)
TNF	39.19 (3.22-375.08)	5.52 (3.9-15.56)	10.66 (3.41-41.7)	6.36 (0.6-43.01)	6.55 (4.66-25.35)	6.73 (1.47-663.69)

Table S4B. Cytokine levels - IFN-γ, IL-12p70, TNF CXCL10, IL-RA, IL-10, IL-1β and IL-6 - in response to phorbol myristate acetate (PMA) plus Ionomycin and tumor necrosis factor (TNF) from 1 - 18 years old (yo). Values are presented as median and interquartile range (Q1–Q3).

Cytokine	Stimulation condition	1-3 yo (N = 9)	>3-5yo (N = 6)	>5-7yo (N = 9)	>7-10yo (N = 14)	>10-14yo (N = 6)	>14-18yo (N = 8)
IFN-γ	SR: PMA-Ionomycin	2050.8 (1590.6-7447.2)	32083.6 (24507.3-40861.7)	3675.0 (669.3-20155.9)	10299.8 (3196.5-30289.1)	26653.7 (8699.0-29305.3)	45431.9 (5758.0-50750.1)
	SR: TNF	1.1 (1.0-1.2)	1.0 (1.0-1.0)	1.0 (0.9-1.2)	1.0 (0.7-1.1)	0.9 (0.6-1.7)	1.0 (0.7-1.0)
IL-10	SR: PMA-Ionomycin	135.2 (51.2-666.5)	1327.8 (580.2-1882.7)	626.1 (79.6-816.7)	922.0 (422.2-4282.7)	1185.9 (947.0-1415.5)	1853.7 (1150.0-2867.9)
	SR: TNF	1.2 (1.1-1.4)	3.8 (1.3-7.1)	1.6 (1.1-5.4)	1.2 (1.1-5.3)	2.1 (1.2-2.6)	1.4 (1.0-3.1)
IL-12p70	SR: PMA-Ionomycin	5.5 (3.5-7.4)	16.2 (13.4-59.2)	3.0 (2.5-6.0)	6.8 (4.0-25.7)	5.1 (3.4-9.6)	28.6 (13.0-41.1)
	SR: TNF	1.0 (0.9-1.1)	0.9 (0.7-1.3)	1.0 (0.4-1.0)	1.0 (1.0-1.6)	1.0 (0.8-1.7)	0.9 (0.6-1.2)
IL-1β	SR: PMA-Ionomycin	134.4 (48.7-246.8)	373.5 (219.1-1322.1)	301.3 (192.7-481.5)	165.5 (44.6-205.5)	552.5 (412.8-759.5)	400.9 (308.7-2390.3)
	SR: TNF	2.9 (1.7-7.9)	1.2 (0.8-1.6)	2.1 (1.4-3.4)	1.0 (0.8-1.2)	2.9 (1.4-3.4)	1.0 (0.6-8.9)

(continued)

(continued)

Cytokine	Stimulation condition	1-3 yo (N = 9)	>3-5yo (N = 6)	>5-7yo (N = 9)	>7-10yo (N = 14)	>10-14yo (N = 6)	>14-18yo (N = 8)
IL-1RA	SR: PMA-Ionomycin	48.5 (24.3-125.9)	70.5 (30.8-90.2)	39.1 (13.7-84.1)	57.0 (16.8-160.1)	102.3 (90.6-115.5)	111.1 (20.7-137.9)
	SR: TNF	3.6 (2.0-22.0)	11.7 (7.1-27.0)	8.0 (3.8-11.4)	11.6 (5.8-28.2)	13.5 (7.1-20.1)	12.0 (7.8-44.7)
IL-6	SR: PMA-Ionomycin	207.5 (90.4-815.7)	1119.4 (559.7-2692.6)	392.2 (291.9-504.8)	278.2 (78.2-346.4)	492.4 (327.2-630.5)	203.1 (170.4-531.4)
	SR: TNF	1.9 (1.2-6.9)	2.7 (1.6-2.9)	4.0 (1.7-6.1)	1.7 (1.3-3.9)	7.0 (3.3-10.7)	1.4 (1.1-11.1)
CXCL10	SR: PMA-Ionomycin	5.1 (2.0-6.5)	4.3 (3.9-4.9)	4.2 (2.4-4.9)	3.6 (2.8-4.0)	4.0 (2.7-4.3)	3.3 (2.3-3.7)
	SR: TNF	1.9 (1.3-3.0)	3.4 (2.1-3.8)	2.7 (1.7-4.7)	1.3 (1.2-4.0)	2.6 (1.9-3.6)	1.5 (1.3-2.3)
TNF	SR: PMA-Ionomycin	307.1 (80.0-437.7)	1271.3 (813.6-1491.6)	599.6 (458.1-1264.3)	820.0 (453.1-1179.9)	1065.7 (921.1-1402.5)	1514.6 (824.3-2758.7)
	SR: TNF	118.6 (44.9-154.7)	1215.6 (435.9-1663.3)	287.6 (163.4-1408.2)	199.4 (69.3-259.7)	127.9 (87.4-301.9)	418.0 (106.5-1501.8)

Abbreviations: SR: stimulation ratio (stimulated condition/baseline condition).

Table S5 IFN-γ receptors (IFN-γR)-1/2 expression in monocytes (CD14+) from 1 - 18 years old (yo). Values are presented as median and interquartile range (Q1–Q3).

Receptor	1-3 yo (N = 9)	>3-5 yo (N = 6)	>5-7 yo (N = 9)	>7-10 yo (N = 14)	>10-14 yo (N = 6)	>14-18 yo (N = 8)
**IFN-γR1 (%)**	73.4 (63.1-83.6)	83.2 (80.2-83.7)	81.6 (30.4-86.7)	59.3 (44.3-82.7)	75.8 (56.3-85.6)	64.8 (49.4-74.5)
**IFN-γR2 (%)**	89.9 (88.3-93.6)	91.0 (89.0-92.9)	89.0 (88.0-93.8)	92.2 (89.4-93.5)	88.9 (88.1-91.8)	82.2 (72.2-91.5)
**IFN-γR1 (MFI)**	1944.0 (498.0-3187.0)	930.5 (681.0-1045.0)	877.0 (494.0-1877.0)	1005.5 (674.2-6274.2)	1494.5 (824.2-1684.8)	967.0 (505.0-1378.5)
**IFN-γR2 (MFI)**	2203.0 (1917.0-2676.0)	2093.0 (1606.8-2319.0)	2221.0 (2062.0-2386.0)	2201.0 (1750.2-2703.0)	1996.5 (1715.0-2650.0)	1809.0 (1499.0-2205.2)

Abbreviations:MFI: mean fluoresnce intensity.

Table S6 Signal transducer and activator of transcription (STAT)-1 phosphorylation and dephosphorylation capacity in a IFN dose dependent manner in monocytes (CD14+) from 1 - 18 years old (yo). Values are presented as median and interquartile range (Q1–Q3).

	Stimulation conditions	1-3 yo (N = 9)	>3-5 yo (N = 6)	>5-7 yo (N = 9)	>7-10 yo (N = 14)	>10-14 yo (N = 6)	>14-18 yo (N = 8)
STAT 1 (%)	10^4 IU/mL hrIFN-γ plus staurosporine (15min)	1.0 (0.7-1.1)	2.3 (1.9-3.2)	1.4 (0.9-3.1)	2.5 (1.1-4.8)	1.5 (1.4-1.6)	1.3 (1.1-2.1)
	10^4 IU/mL hrIFN-γ plus staurosporine (30min)	0.9 (0.3-1.5)	1.0 (0.9-1.2)	1.2 (0.7-3.0)	1.9 (1.1-3.2)	0.9 (0.7-1.1)	0.8 (0.7-2.1)
	10^2 IU/mL hrIFN-γ	1.7 (0.5-2.0)	2.3 (1.5-4.7)	1.9 (1.4-3.2)	3.7 (2.5-4.9)	2.9 (1.4-3.7)	3.0 (2.1-4.8)
	10^4 IU/mL hrIFN-γ	2.8 (2.3-4.9)	4.9 (3.4-6.0)	3.8 (1.7-6.4)	4.7 (3.2-7.7)	4.3 (3.7-5.7)	6.4 (4.9-7.9)
	10^3 IU/mL IFN-α	2.7 (1.5-2.8)	2.7 (2.0-4.5)	3.3 (1.8-5.9)	3.5 (1.9-5.6)	2.3 (1.2-3.4)	3.2 (2.1-4.4)
STAT 1 (MFI)	10^4 IU/mL hrIFN-γ plus staurosporine (15min)	1.1 (0.9-1.4)	1.4 (1.2-3.0)	1.4 (1.1-2.8)	1.9 (1.1-2.8)	1.7 (1.1-2.3)	1.9 (1.6-3.6)
	10^4 IU/mL hrIFN-γ plus staurosporine (30min)	1.0 (1.0-1.3)	1.2 (1.1-2.3)	1.1 (1.0-1.9)	1.9 (1.0-2.7)	1.4 (1.1-1.5)	1.6 (1.4-3.7)
	10^2 IU/mL hrIFN-γ	1.5 (1.1-1.7)	1.5 (1.1-5.4)	1.3 (1.2-2.6)	2.0 (1.2-2.8)	1.9 (1.0-3.1)	2.2 (1.7-4.9)
	10^4 IU/mL hrIFN-γ	1.6 (1.4-2.2)	1.6 (1.6-6.1)	1.6 (1.4-4.8)	2.4 (1.2-3.7)	2.6 (1.2-4.5)	2.5 (1.9-7.3)
	10^3 IU/mL IFN-α	1.4 (1.3-2.4)	1.6 (1.4-5.1)	1.5 (1.3-4.7)	1.9 (1.2-3.0)	1.7 (1.1-3.3)	2.3 (1.7-4.0)

Abbreviations:MFI: mean fluoresnce intensity. The results are stimulation rations: stimulated condition /baseline condition.

Table S7 T cell subsets frequency (%) from 1 - 18 years old (yo). Values are presented as median and interquartile range (Q1–Q3).

T cell subsets	From	1-3 yo (N = 9)	>3-5yo (N = 6)	>5-7yo (N = 9)	>7-10yo (N = 14)	>10-14yo (N = 6)	>14-18yo (N = 8)
CD3+ **T cell**	Total lymphocyte	62.8 (61.5-65.5)	68.8 (62.7-69.8)	62.2 (59.0-66.8)	63.8 (60.5-67.4)	65.4 (60.6-68.2)	74.0 (68.8-78.7)
CD3+TCR αβ **TCR αβ**	CD3+	88.3 (86.0-90.1)	90.5 (84.8-92.9)	91.5 (87.1-93.4)	87.5 (84.0-88.8)	91.5 (87.4-91.9)	90.8 (86.9-91.9)
CD3+TCR γδ **TCR γδ**	CD3+	8.8 (7.6-10.4)	7.8 (6.0-12.0)	7.7 (5.6-10.3)	10.4 (9.6-13.7)	7.0 (6.3-11.3)	7.0 (6.4-11.3)
CD3+CD4+	Total lymphocyte	35.4 (32.2-39.9)	34.0 (26.6-39.4)	34.1 (32.1-37.8)	32.5 (29.1-35.6)	36.5 (32.5-40.5)	33.3 (28.8-40.2)
CD3+CD4+CD45RA-CCR7+ **Central memory Th**	CD4+	12.4 (8.6-13.7)	11.0 (9.8-13.8)	15.0 (13.5-16.6)	17.4 (14.7-23.8)	19.6 (18.1-25.2)	25.8 (18.1-31.5)
CD3+CD4+CD45RA+CD45RO+ **Early memory Th**	CD4+	2.9 (2.1-3.5)	4.7 (3.1-7.3)	2.1 (1.3-3.7)	3.1 (2.6-4.0)	3.7 (2.3-4.2)	3.6 (2.7-4.1)
CD3+CD4+CD45RA+CCR7- **Effector memory RA Th**	CD4+	2.3 (1.6-4.2)	2.8 (2.5-3.7)	1.9 (1.7-2.9)	1.8 (1.6-2.2)	2.9 (1.9-4.2)	2.5 (1.6-3.0)
CD3+CD4+HLA-DR+ **Activated Th**	CD4+	3.7 (3.1-5.1)	6.3 (4.8-8.2)	5.5 (4.8-6.4)	6.6 (4.6-7.3)	5.7 (5.1-7.2)	4.9 (4.3-7.3)

Abbreviations: CCR: CC chemokine receptors; CXCR: C-X-C chemokine receptor; TCR: T cell receptor; Tfh: T follicular helper.

(continued)

(continued)

T cell subsets	From	1-3 yo (N = 9)	>3-5yo (N = 6)	>5-7yo (N = 9)	>7-10yo (N = 14)	>10-14yo (N = 6)	>14-18yo (N = 8)
CD3+CD8+	Total lymphocyte	22.1 (20.0-23.9)	23.5 (22.9-24.3)	23.0 (19.4-26.5)	22.6 (20.7-25.8)	21.1 (19.0-22.9)	28.7 (27.9-31.5)
CD3+CD8+CD45RA+CCR7+ **Naïve Tc**	CD8+	66.9 (55.6-76.7)	53.0 (33.3-65.6)	47.9 (42.2-60.9)	49.0 (37.4-57.6)	45.6 (38.4-53.3)	43.9 (39.1-48.5)
CD3+CD8+CD45RA-CCR7+ **Central memory Tc**	CD8+	2.8 (1.9-3.6)	3.3 (1.6-3.7)	2.9 (2.5-3.1)	4.6 (3.6-6.0)	5.3 (4.3-6.0)	2.5 (1.8-3.6)
CD3+CD8+CD45RA-CCR7- **Effector memory Tc**	CD8+	14.8 (10.9-21.2)	21.1 (19.5-27.3)	26.1 (20.1-31.1)	34.7 (26.1-42.4)	36.0 (28.1-41.8)	25.1 (22.1-26.8)
CD3+CD8+CD45RA+CCR7- **Effector memory RA Tc**	CD8+	14.6 (7.4-23.7)	16.0 (11.6-33.0)	18.5 (15.6-20.8)	12.1 (7.6-14.9)	11.7 (8.4-15.8)	29.8 (15.7-38.9)
CD3+CD8+HLA-DR+ **Activated Tc**	CD8+	9.4 (4.3-16.7)	14.9 (11.4-15.9)	14.6 (9.9-22.6)	12.8 (7.3-17.1)	13.6 (10.6-15.4)	16.0 (13.6-21.0)
CD3+CD4-CD8- cells	Total lymphocyte	5.0 (4.3-7.5)	3.9 (3.1-5.3)	3.3 (2.8-5.0)	5.5 (3.6-6.3)	2.4 (2.1-3.8)	4.9 (3.0-5.6)
CD3+CD4-CD8- cells	TCR αβ	1.7 (1.4-1.8)	1.5 (1.3-1.6)	1.4 (1.1-1.7)	1.5 (1.2-1.8)	1.4 (1.3-1.5)	1.5 (1.2-2.4)
CD3+CD4+CD57+ **Terminally differentiated Th**	CD4+	2.0 (1.7-2.3)	1.7 (1.6-5.0)	2.3 (1.8-4.3)	3.3 (2.3-3.7)	2.7 (1.6-3.7)	3.8 (3.1-5.5)
CD3+CD4+CD69+ **Activated Th**	CD4+	0.7 (0.5-0.8)	1.6 (1.3-1.9)	1.0 (0.8-1.4)	0.8 (0.7-0.9)	1.3 (1.1-2.2)	1.8 (1.4-2.2)
CD3+CD8+CD57+ **Terminally differentiated Tc**	CD8+	10.5 (6.9-21.8)	19.9 (11.3-34.8)	25.0 (20.2-38.8)	22.9 (10.5-27.9)	21.4 (11.5-29.9)	33.2 (25.5-34.4)
CD3+CD8+CD69+ **Activated Tc**	CD8+	0.5 (0.3-0.9)	0.4 (0.3-0.7)	0.5 (0.4-0.5)	0.4 (0.3-0.5)	0.3 (0.2-0.7)	0.4 (0.3-0.7)

Table S8 T cell proliferative capacity from 1 - 18 years old (yo). Values are presented as median and interquartile range (Q1–Q3). Stimulation conditions included phytohemaglutinin A (PHA:), pokeweed mitogen (PWM) and Concanavalin A (ConA). Division index (DI) and proliferation index (DI) were calculated from lymphocyte and T cells (CD3+).

	**Stimulation condition**	1-3 yo (N = 9)	>3-5yo (N = 6)	>5-7yo (N = 9)	>7-10yo (N = 14)	>10-14yo (N = 6)	>14-18yo (N = 8)
**DI_Lym**	**ConA** (2 µg/mL)	15.6 (11.2-21.0)	5.2 (5.1-6.7)	7.5 (4.5-60.9)	18.3 (5.6-176.8)	13.6 (8.9-24.5)	3.6 (3.5-4.4)
	**PHA** (5µg/mL)	9.6 (5.4-12.8)	2.4 (2.2-2.6)	6.5 (2.4-37.8)	4.4 (2.8-251.0)	7.6 (7.1-8.9)	2.3 (1.5-3.5)
	**PWM** (2 µg/mL)	5.1 (2.0-6.7)	1.7 (1.1-5.0)	3.4 (1.7-6.4)	7.9 (2.5-80.9)	1.8 (1.3-8.6)	1.4 (1.1-2.0)
**DI_Tcell**	**ConA** (2 µg/mL)	53.2 (18.3-133.6)	59.4 (42.2-113.3)	58.0 (7.0-146.9)	133.5 (19.4-286.8)	10.8 (7.9-64.3)	7.2 (6.2-9.6)
	**PHA** (5µg/mL)	40.0 (9.3-50.3)	39.4 (16.3-44.4)	25.8 (6.6-77.0)	94.6 (8.2-403.0)	6.6 (4.2-16.7)	6.0 (2.4-12.0)
	**PWM** (2 µg/mL)	10.5 (2.1-28.5)	6.7 (6.6-47.8)	17.4 (2.4-38.6)	14.6 (5.1-79.8)	2.1 (0.6-6.8)	3.5 (1.7-4.5)
**PI_Lym**	**ConA** (2 µg/mL)	1.0 (0.9-1.1)	1.1 (1.1-1.2)	1.6 (1.1-2.0)	1.3 (0.9-1.8)	1.6 (1.5-2.1)	1.2 (1.0-1.7)
	**PHA** (5µg/mL)	0.9 (0.7-1.2)	1.1 (1.0-1.2)	1.4 (0.8-1.7)	1.1 (0.9-1.7)	1.5 (1.5-1.5)	0.9 (0.8-1.0)
	**PWM** (2 µg/mL)	1.2 (1.2-1.8)	1.7 (1.5-1.8)	1.6 (1.1-2.4)	1.4 (0.9-2.3)	1.7 (1.7-2.0)	1.5 (1.4-1.9)
**PI_Tcell**	**ConA** (2 µg/mL)	1.7 (1.5-1.8)	1.5 (1.2-1.6)	1.7 (1.4-2.0)	1.6 (1.1-2.1)	1.8 (1.4-2.0)	1.6 (1.2-1.9)
	**PHA** (5µg/mL)	1.4 (1.1-1.6)	1.1 (0.9-1.3)	1.5 (1.0-1.9)	1.3 (1.0-1.7)	1.5 (1.2-1.8)	1.5 (1.1-1.6)
	**PWM** (2 µg/mL)	2.4 (1.7-3.0)	1.6 (1.4-2.4)	2.2 (1.7-3.0)	1.4 (1.2-2.9)	1.5 (1.4-1.8)	2.0 (1.9-2.1)

SUPPLEMENTARY FIGURES

Figure S1 Graphical summary of the methods employed for immune assays.

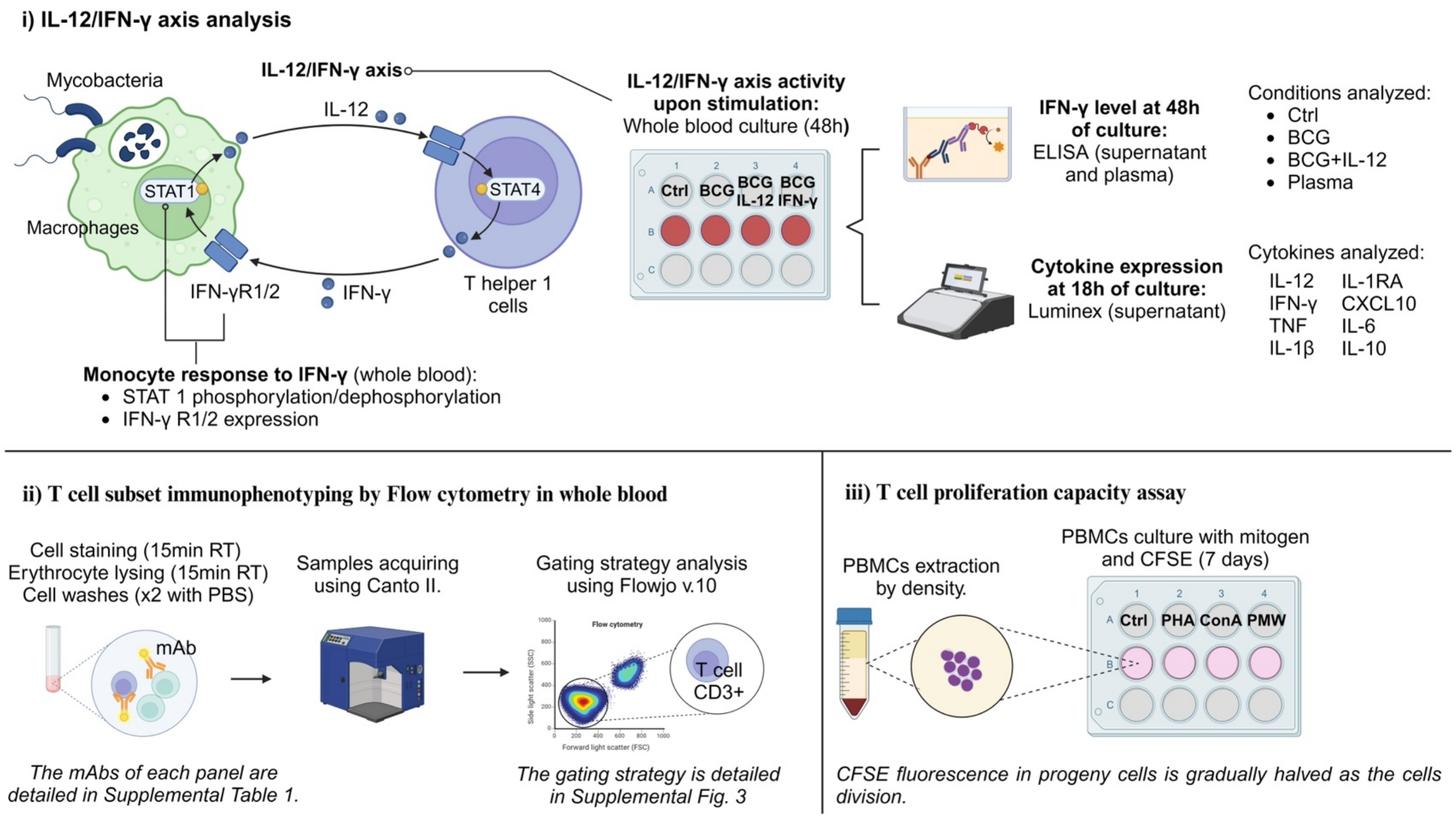

Abbreviations: BCG: Bacillus Calmette-Guérin; CFSE: carboxyfluorescein succinimidyl ester; ConA: concavalin A; C-X-C ligand 10; ELISA: enzyme-linked immunosorbent assay; IFN: interferon; IFN-γR: IFN-γ receptors; IL: interleukin; IL-1RA: IL-1 receptor antagonist; mAb: monoclonal antibodies; PBMC: peripheral blood mononuclear cells; PBS: phosphate-buffered saline; PHA: phytohemagglutinin; PMW: pokeweed; RT: room temperature; STAT: signal transducer and activator of transcription. This figure is original and created by Y. Luo in Biorender.com.

Figure S2 IFN-γ receptor (IFN-γR) 1 and 2 expression gating strategy. The expression of IFN-γR1 (CD119) and IFN-γR2 was gated from monocytes. The unstained condition was used to identify the negative population (DN: double negative for both receptors) appropriately. The mean fluorescence intensity (MFI) was also evaluated for both receptors.

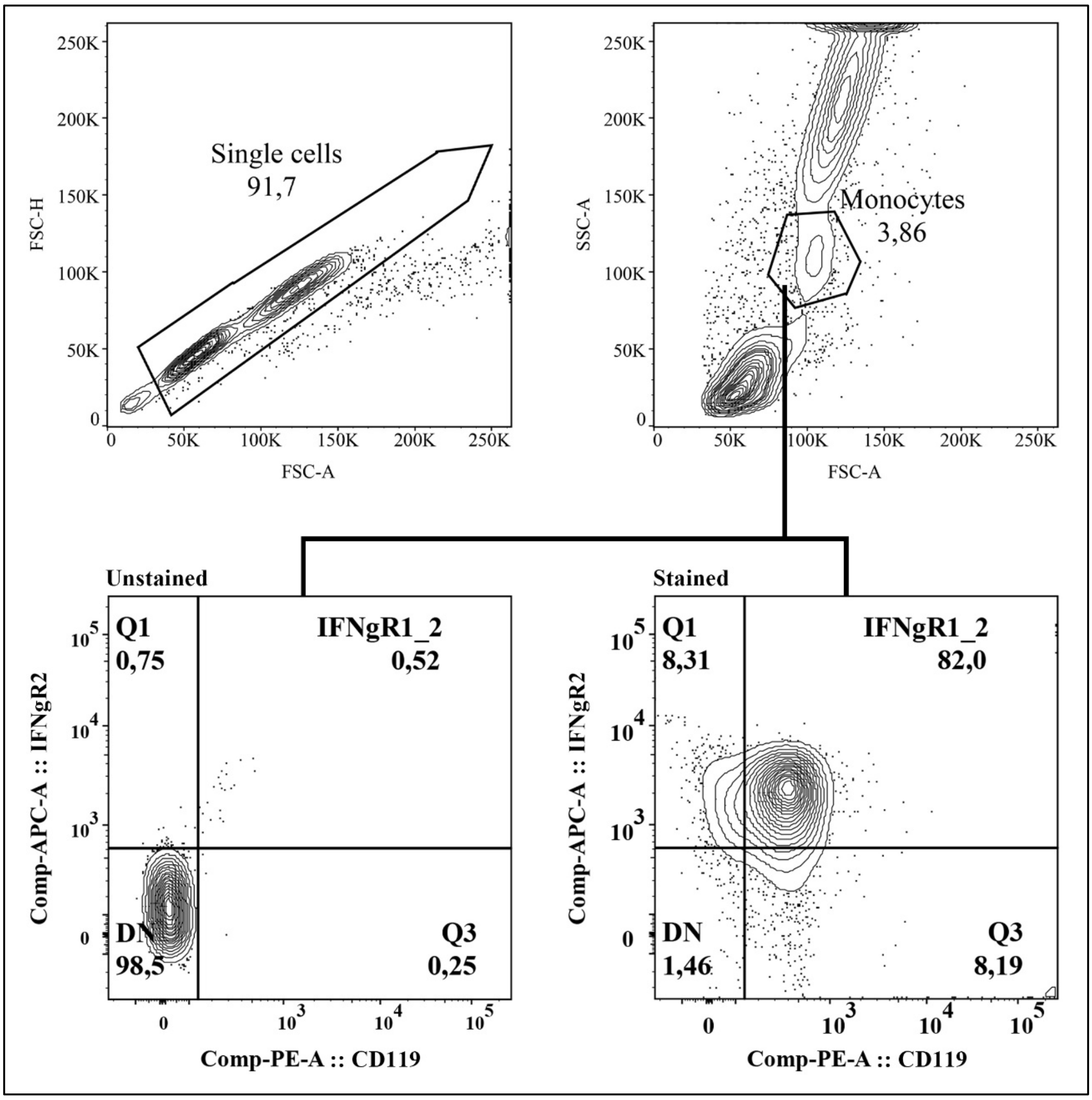

This figure is original, exported from Flowjo v.10 and layouted using Adobe Photoshop.

Figure S3 Signal transducer and activator of transcription (STAT1) 1 phosphorylation and dephosphorylation capacity gating strategy. The expression of STAT1 was gated from monocytes ($CD14^+$). The stimulation conditions were 1) 10^2 IU/mL hrIFN-γ; 2) 10^4 IU/mL hrIFN-γ; 3) 10^4 IU/mL hrIFN-γ followed by washing and incubation with staurosporine for 15 minutes; 4) 10^4 IU/mL hrIFN-γ with staurosporine for 30 minutes; 5) 10^3 IU/mL IFN-α. The mean fluorescence intensity (MFI) was also evaluated for STAT1.

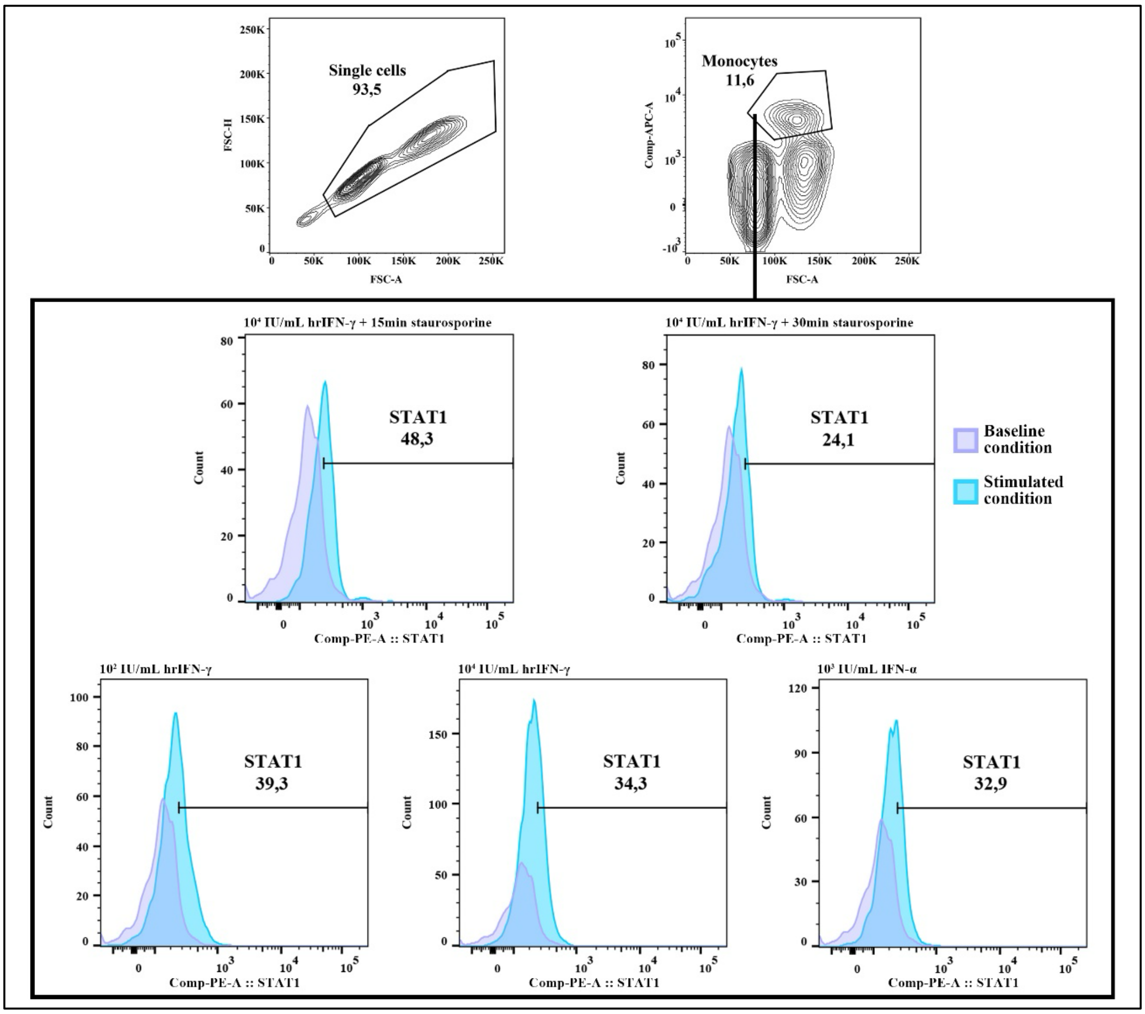

This figure is original, exported from Flowjo v.10 and layouted using Adobe Photoshop

Figure S4 T cell compartment immunophenotyping gating strategy.

The contour plot TCRγδ (FITC) vs TCRαβ (PE) was highlighted to illustrate fluorescence distribution and confirm absence of autofluorescence interference. The absence of unspecific background signal in the double-negative and single-positive quadrants supports minimal autofluorescence interference in FITC and PE channels. Quadrant statistics (%) are displayed, confirming the reliability of fluorescence detection in whole blood cytometry without excessive background noise.

(Continued on next page)

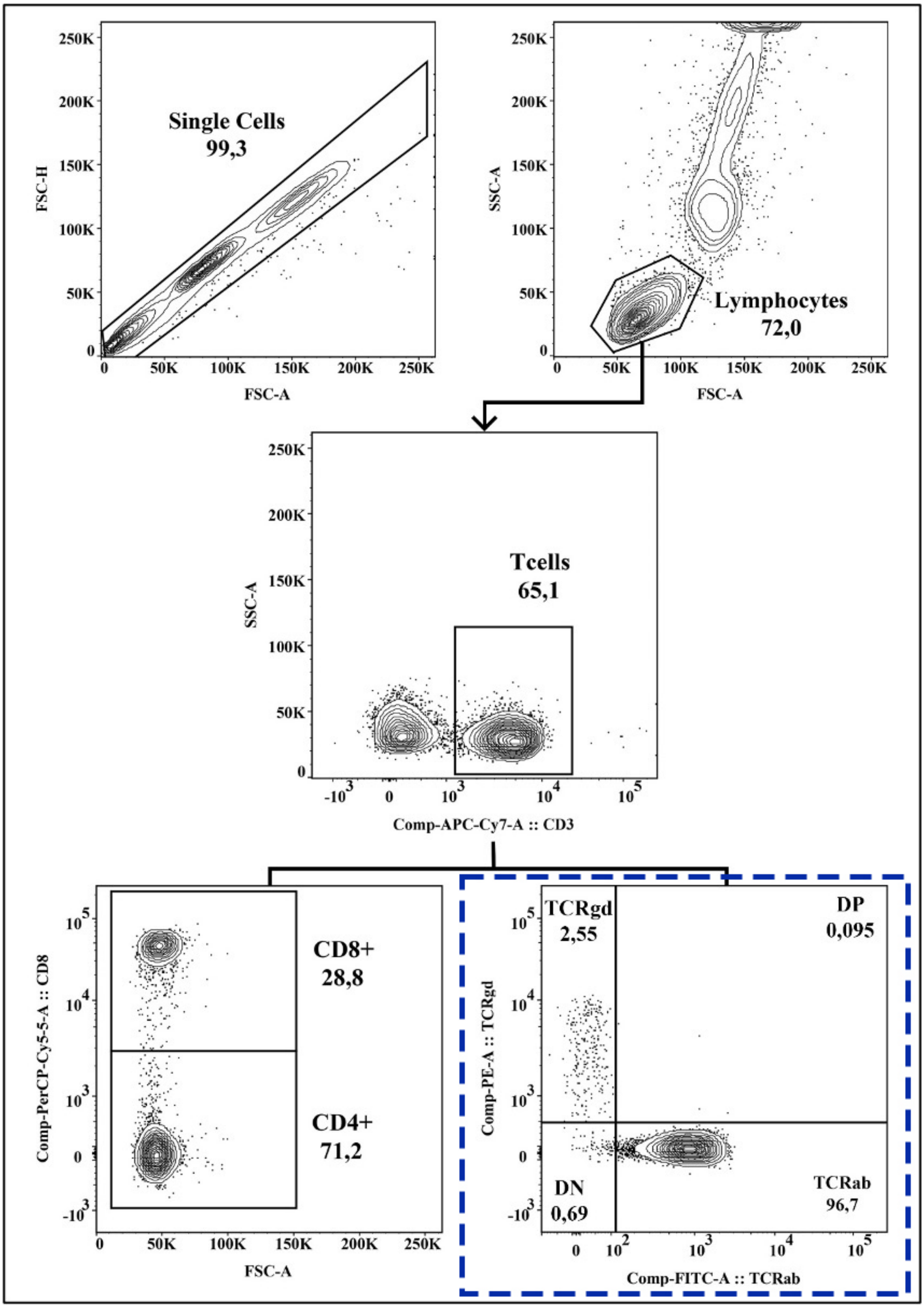

(Continued on next page)

(continued from CD4+ and CD8+ cells)

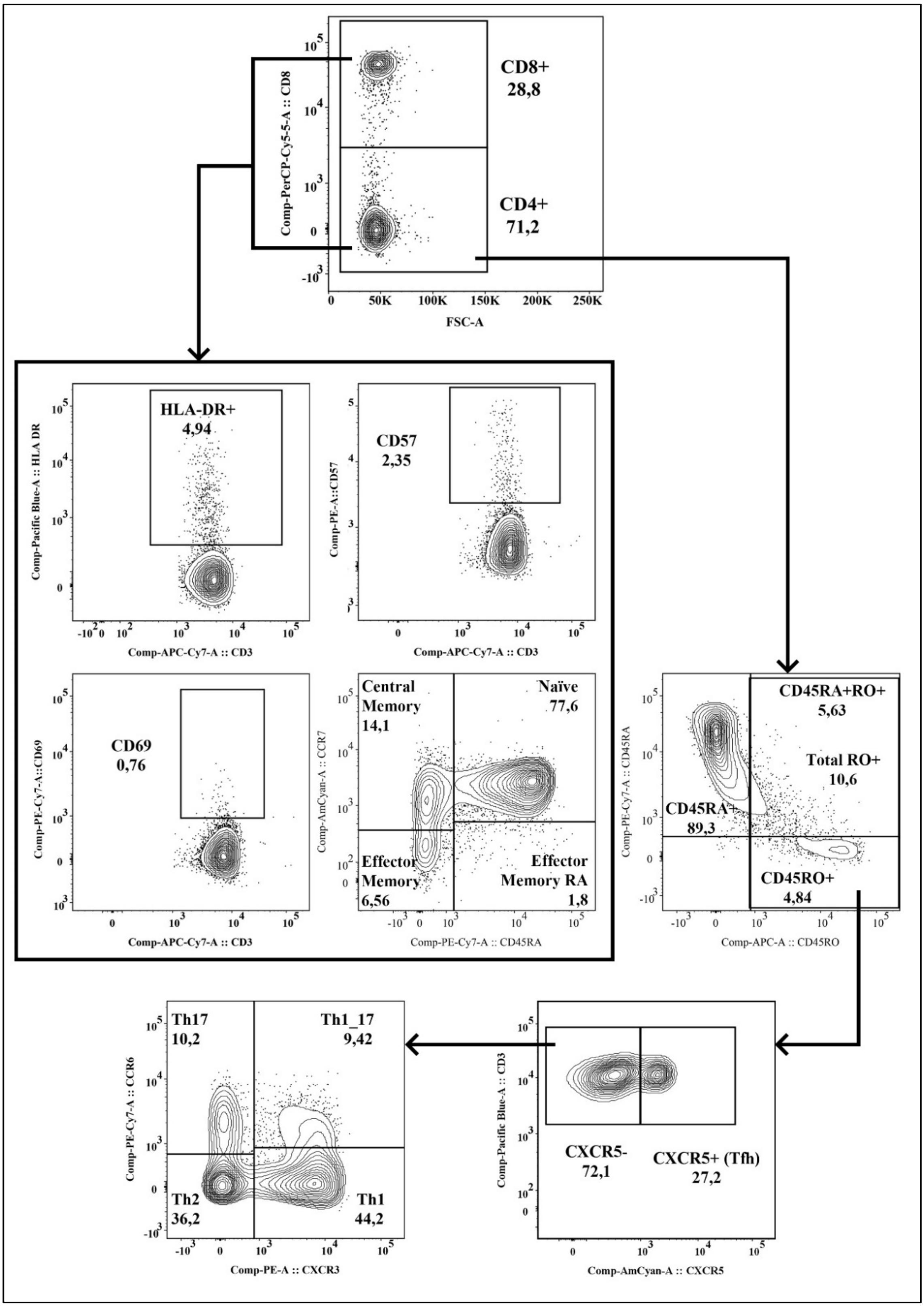

This figure is original, exported from Flowjo v.10 and layouted using Adobe Photoshop

Figure S5 Proliferation assay gating strategy. Stimulation conditions included negative control (baseline condition), phytohemaglutinin A (PHA), pokeweed mitogen (PWM) and Concanavalin A (ConA). Division index (DI) and proliferation index (DI) were calculated from lymphocyte and T cells (CD3+) after 7 days of culture.

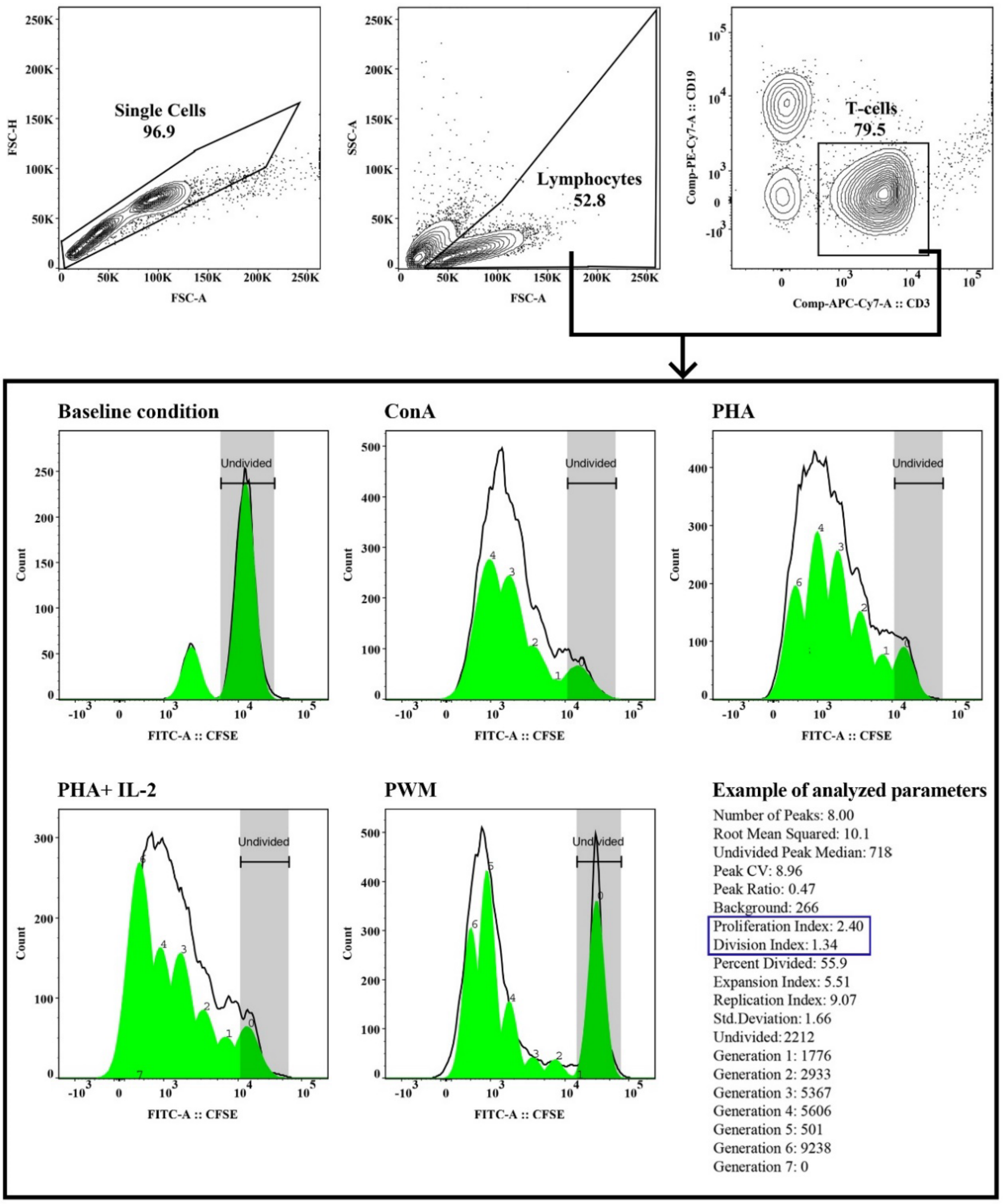

Each peak (Arabic number) represents the Generation of cell division; for example, the Generation 0 peak represents the undivided cells (baseline condition). The optimal Peak Ratio was manually adjusted and fixed in Flowjo to set a Peak Ratio close to the expected 0.5 percent (it is expected that each time a cell divides, each of the two cells in the next generation will have half as much dye (CFSE: carboxifluorescein diacetate succinimidyl ester), so their signal will be half as intense. This figure is original, exported from Flowjo v.10 and layouted using Adobe Photoshop

Figure S6 Correlation of IFN-γ levels with age and T helper (Th) cells subsets. IFN-γ levels were assessed with ELISA after 48h of culture. Stimulation conditions included: baseline levels in plasma, and in response to Bacille Calmette-Guérin (BCG), BCG plus IL-12, and phorbol myristate acetate (PMA) plus Ionomycin.

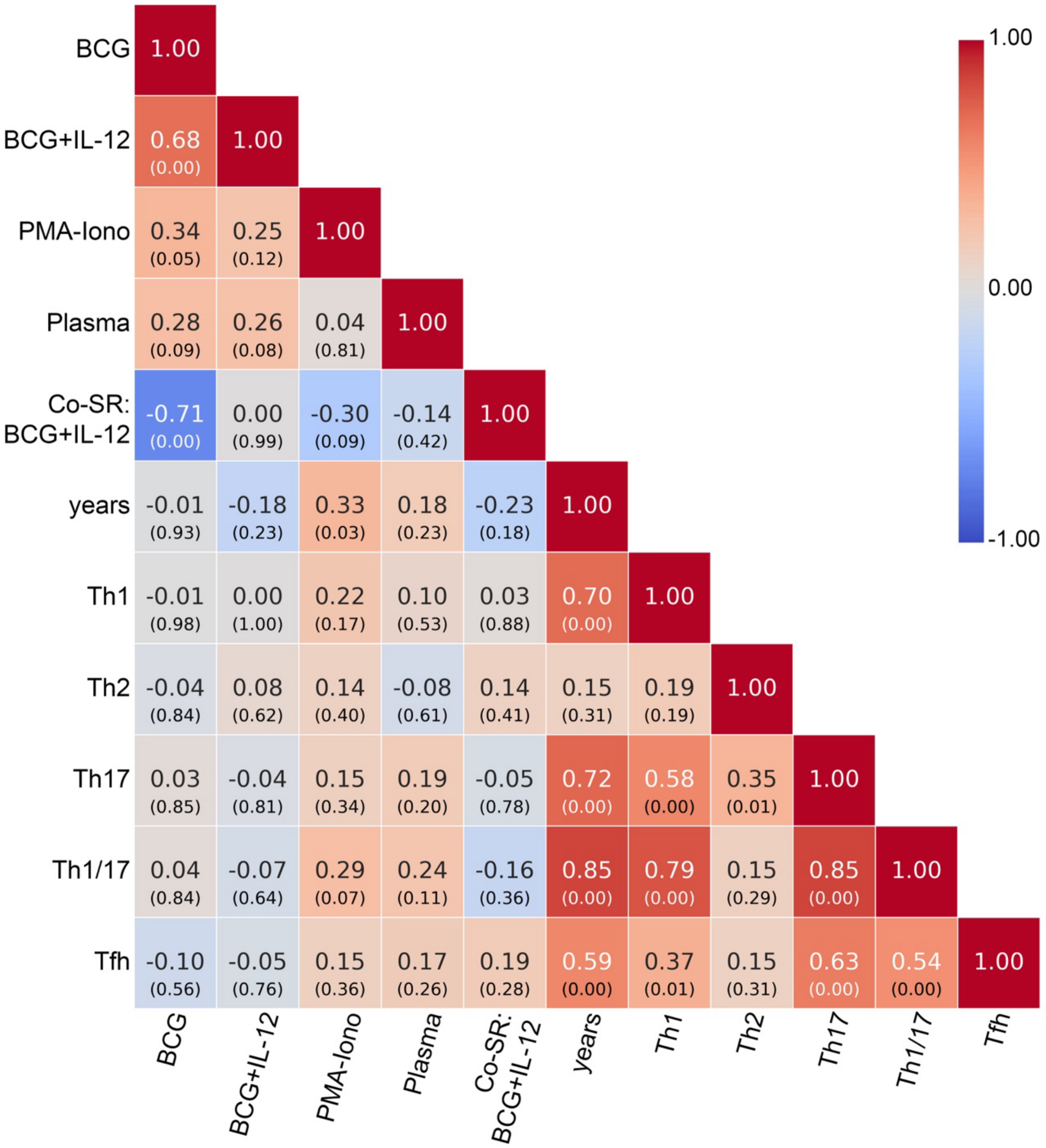

For the stimulations BCG, BCG plus IL-12 and PMA plus Ionomycin we calculated the stimulation ratio (SR: stimulated condition/basal condition). For BCG plus IL-12 we also calculated the Co-SR (BCG plus IL-12/BCG). Abbreviations: Tfh: T follicular helpe.. Spearman correlation (r): low association 0.1-0.3; moderate positive association between 0.3-0.5; and strong positive association 0.5-1. Statistical significance: $p < 0.05$.

Figure S7 Correlation of cytokine levels - IFN- γ, IL-10, IL-12p70, IL-1β, IL-1RA, IL-6, CXCL10 (IP-10), TNF - with age and T helper (Th) cells subsets. Cytokine levels were assessed by Luminex after 18h of culture. Stimulation conditions included: Bacille Calmette-Guérin (BCG), BCG plus IL-12, and BCG plus IFN-γ.

A) Stimulation conditions (SR: stimulated condition/basal condition) for each cytokine.

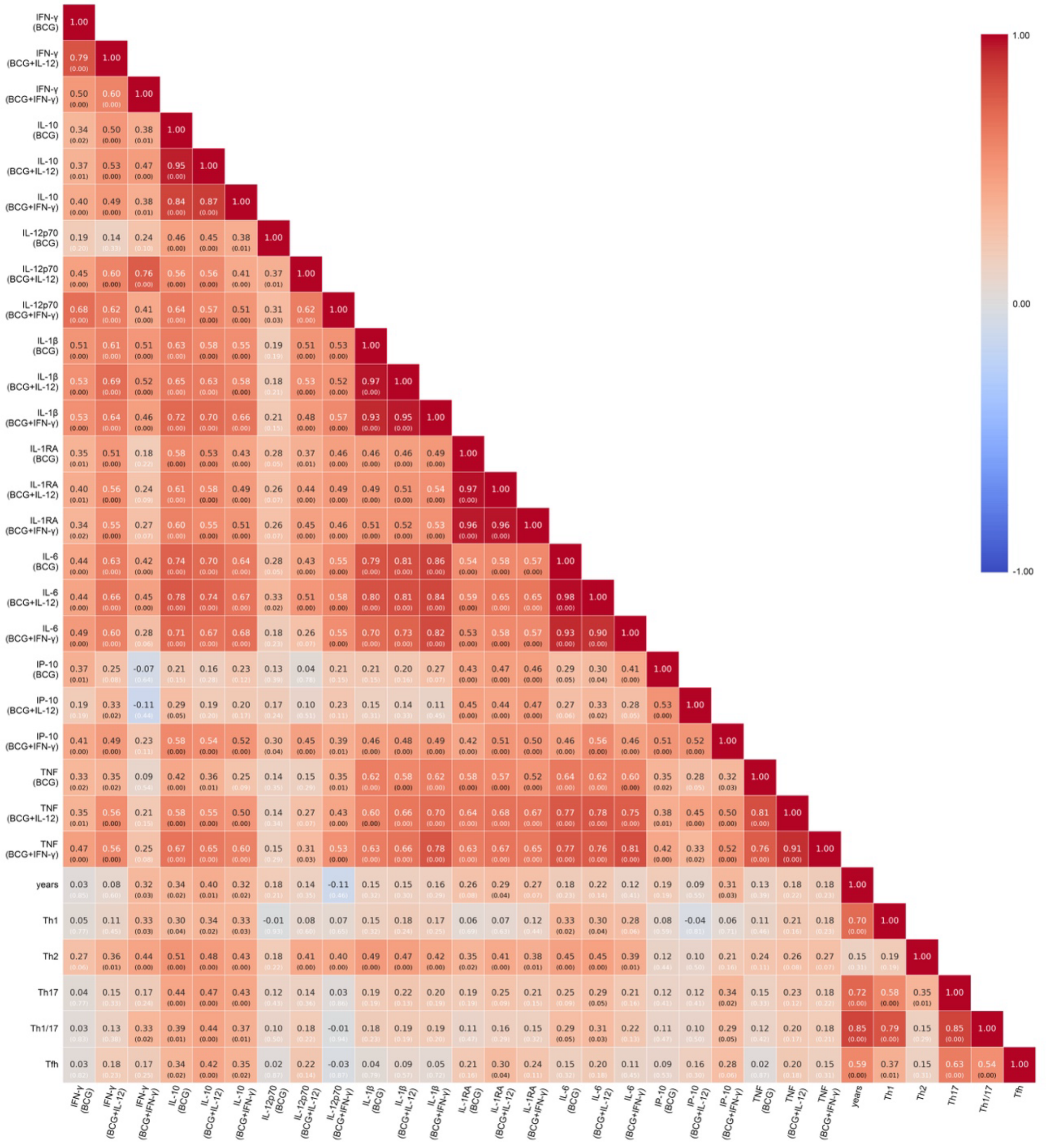

B) Co-stimulation ratio (Co-SR: stimulated condition/BCG) for each cytokine.

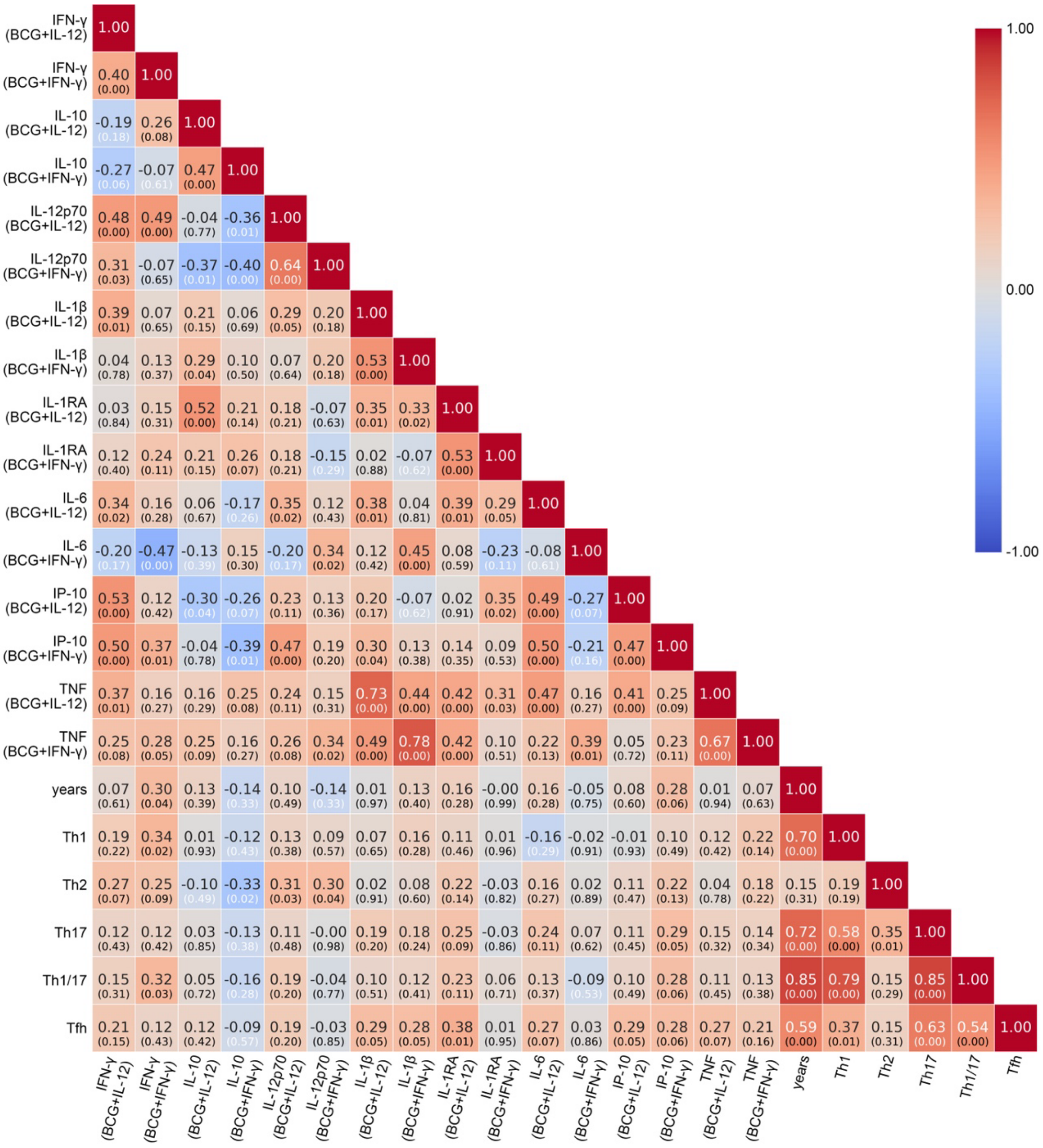

Abbreviations: Tfh: T follicular helper. Spearman correlation (r): low association 0.1-0.3; moderate positive association between 0.3-0.5; and strong positive association 0.5-1. Statistical significance: $p < 0.05$.

Figure S8 Correlation of IFN-γ receptors (IFN-γR)-1/2 with age and T helper (Th) cells subsets.

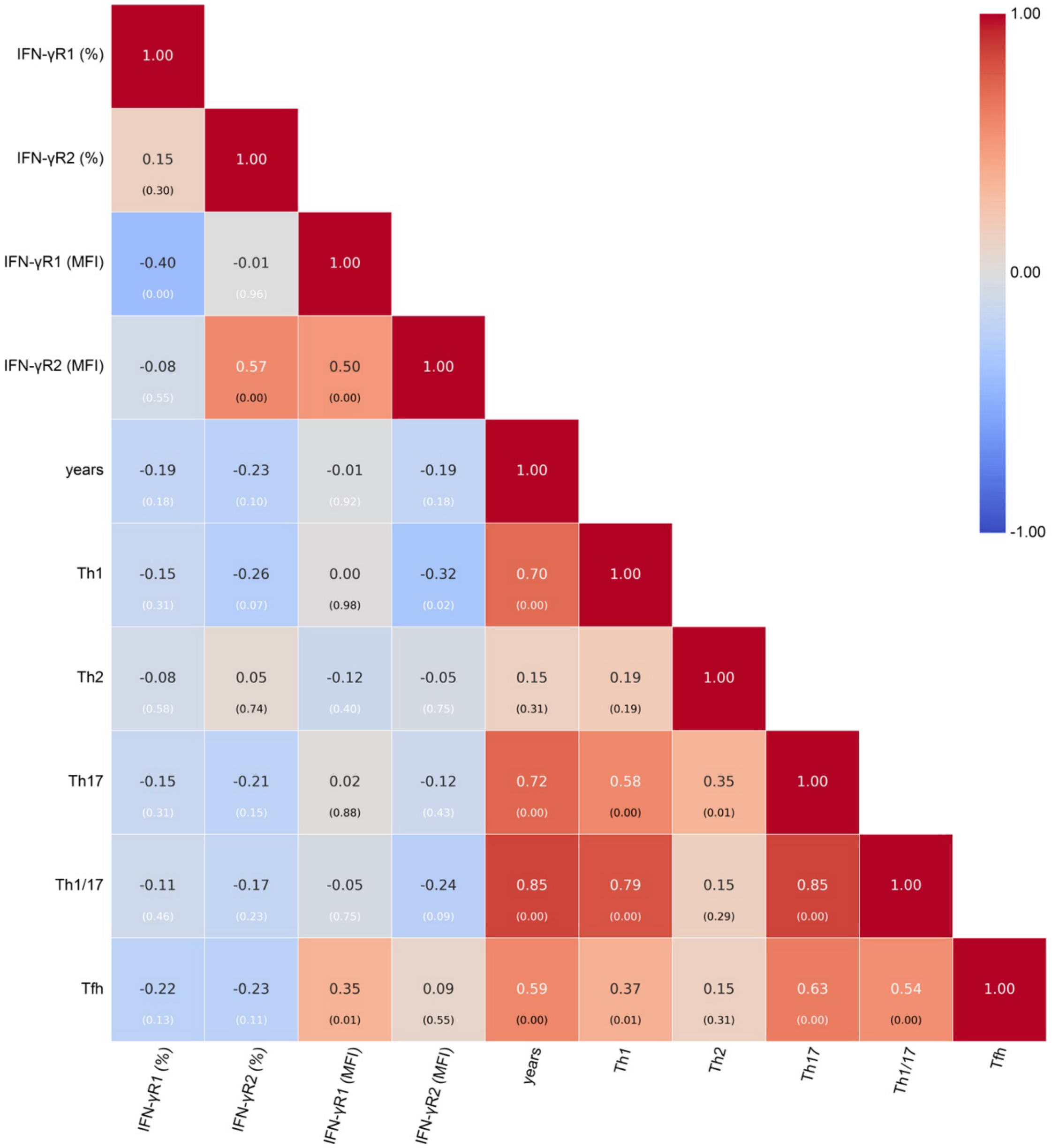

Abbreviations: Tfh: T follicular helper; MFI: mean fluorescence intensity. Spearman correlation (r): low association 0.1-0.3; moderate positive association between 0.3-0.5; and strong positive association 0.5-1. Statistical significance: p < 0.05.

Figure S9 Correlation of Signal transducer and activator of transcription (STAT)-1 with age and T helper (Th) cells subsets. The stimulation conditions included: 1) 10^2 IU/mL hrIFN-γ; 2) 10^4 IU/mL hrIFN-γ; 3) 10^4 IU/mL hrIFN-γ followed by washing and incubation with staurosporine for 15 min; 4) 10^4 IU/mL hrIFN-γ followed by washing and incubation with staurosporine for 30 min; and 5) 10^3 IU/mL IFN-α

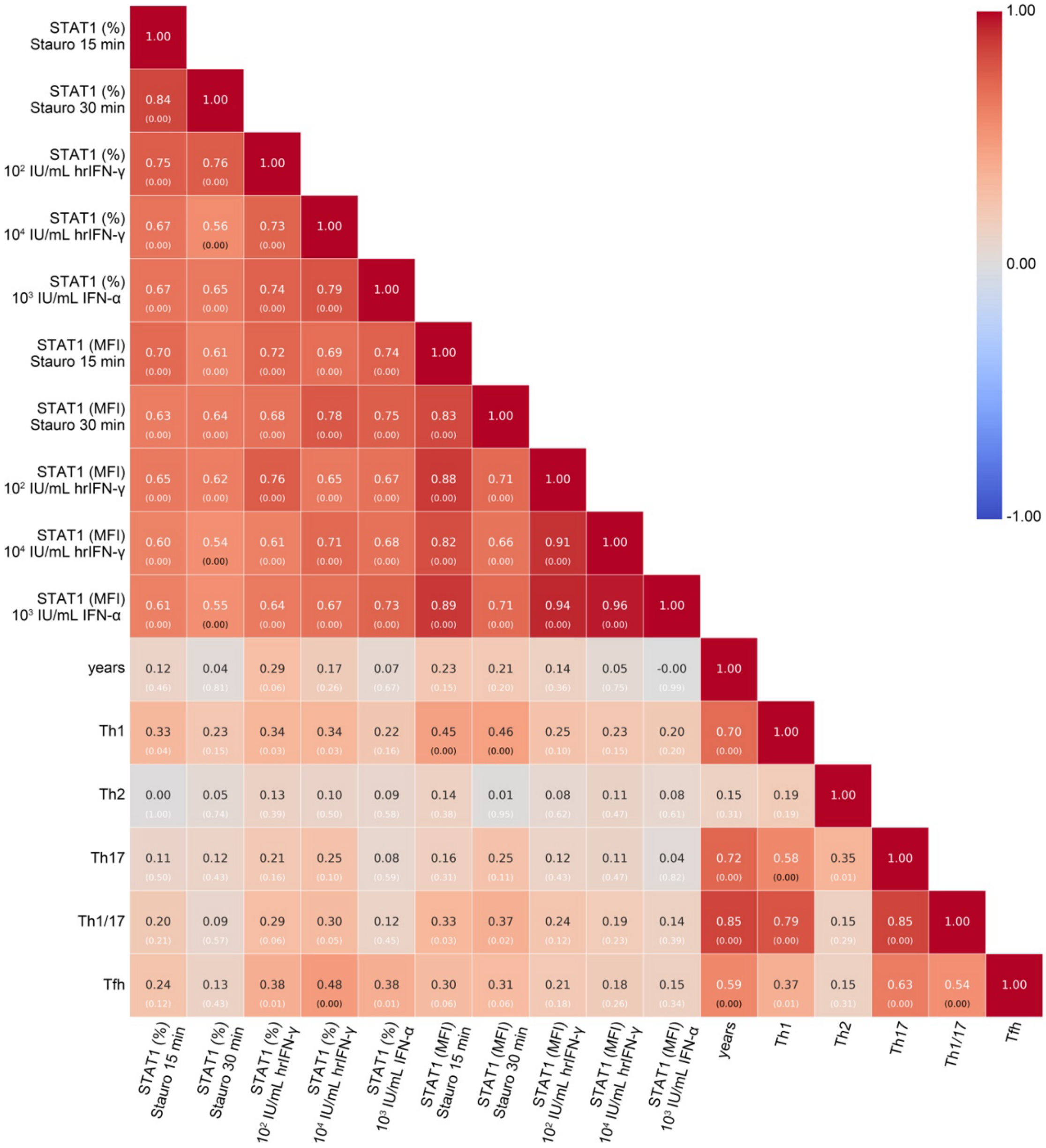

Abbreviations: Tfh: T follicular helper; MFI: mean fluorescence intensity. Spearman correlation (r): low association 0.1-0.3; moderate positive association between 0.3-0.5; and strong positive association 0.5-1. Statistical significance: $p < 0.05$.

Figure S10 Correlation of T cell subsets with age.

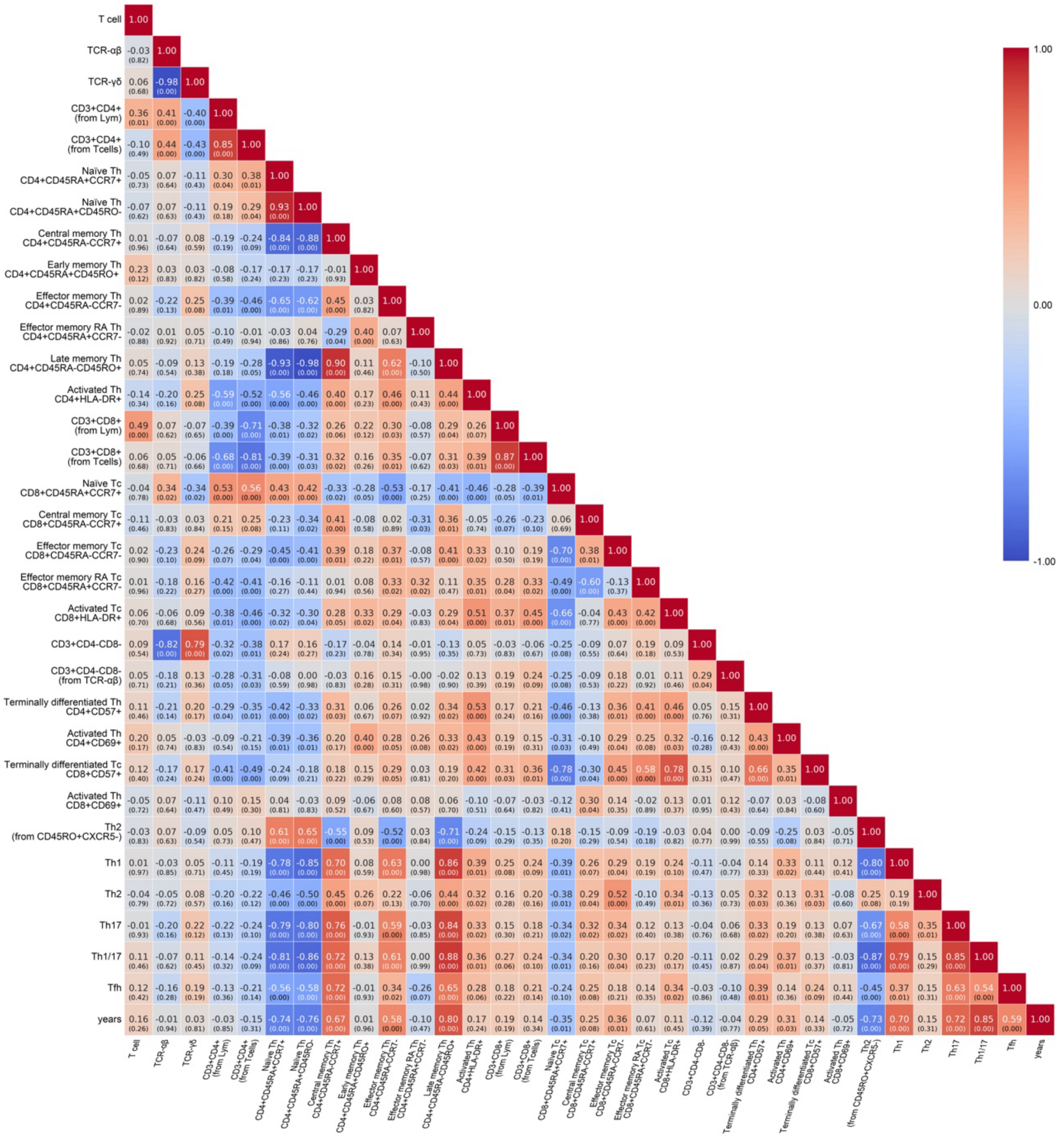

Abbreviations: CCR: CC chemokine receptors; CXCR: C-X-C chemokine receptor; TCR: T cell receptor; Tfh: T follicular helper. Spearman correlation (r): low association 0.1-0.3; moderate positive association between 0.3-0.5; and strong positive association 0.5-1. Statistical significance: $p < 0.05$.

Figure S11 Correlation of T cell subsets defined with CD45RA/CCR7 vs CD45RA/CD45RO.

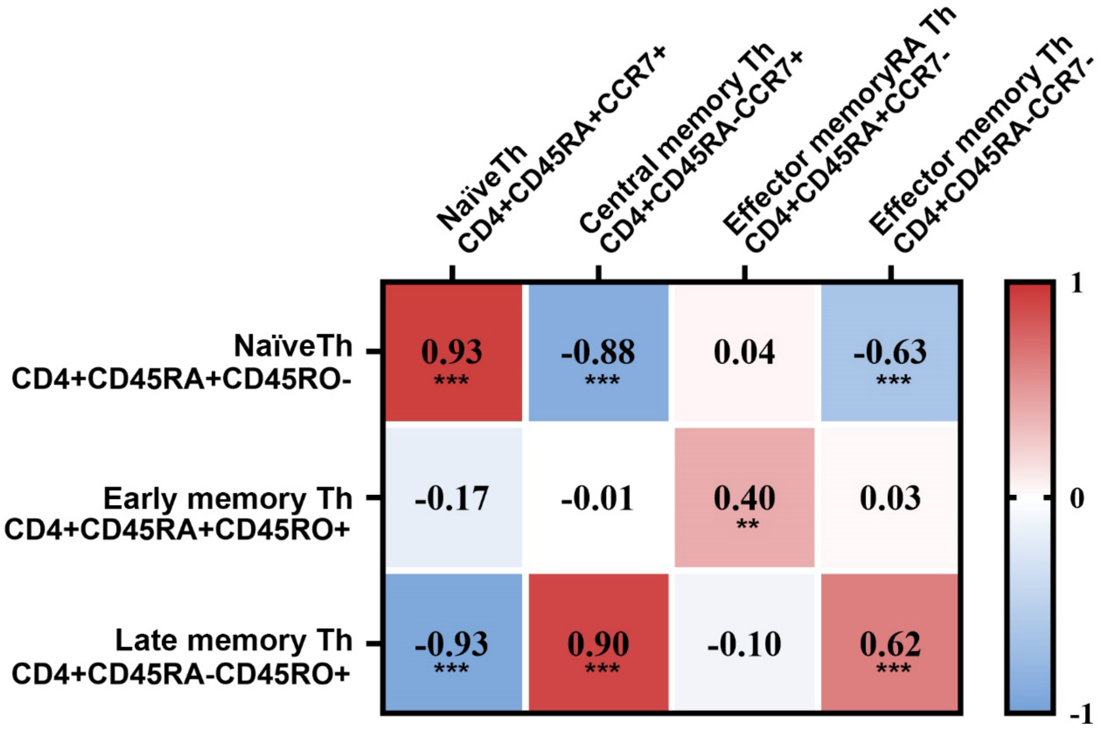

*Abbreviations: CCR: CC chemokine receptors; Th: T helper. Statistical significance p < 0.05; *: p < 0.05; **: p < 0.01; ***: p < 0.001. Spearman correlation (r): low association 0.1-0.3; moderate positive association between 0.3-0.5; and strong positive association 0.5-1.*

Figure S12 Correlation of T cell proliferative capacity with age and T helper (Th) cells. Stimulation conditions included negative control (baseline condition), phytohemaglutinin A (PHA), pokeweed mitogen (PWM) and Concanavalin A (ConA). Division index (DI) and proliferation index (DI) were calculated from lymphocyte and T cells (CD3+) after 7 days of culture.

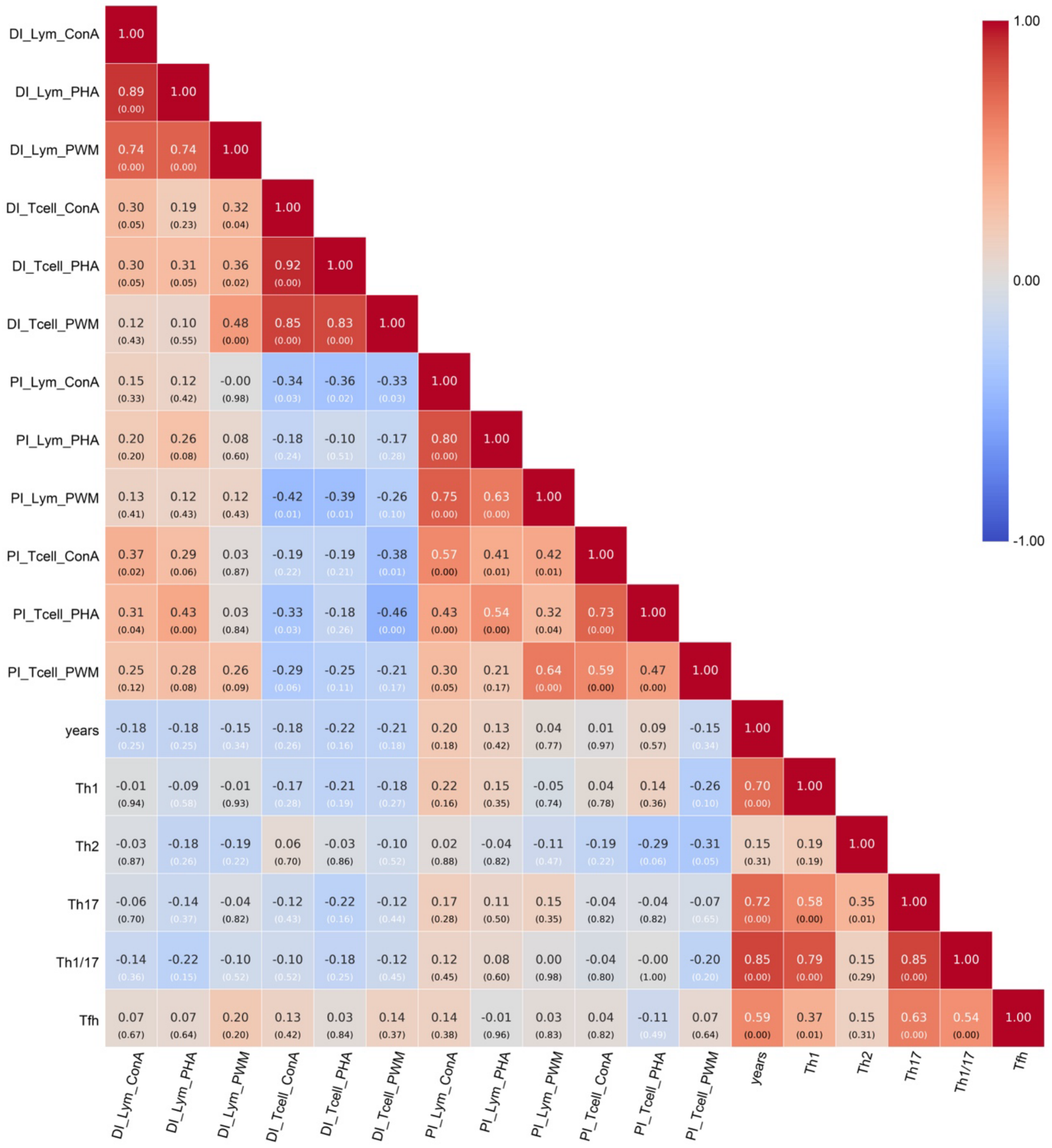

Abbreviations: Tfh: T follicular helper. Spearman correlation (r): low association 0.1-0.3; moderate positive association between 0.3-0.5; and strong positive association 0.5-1. Statistical significance: $p < 0.05$.

SUPPLEMENTARY REFERENCES

1. Esteve-Solé A, Sologuren I, Martínez-Saavedra MT, Deyà-Martínez À, Oleaga-Quintas C, Martinez-Barricarte R, et al. Laboratory evaluation of the IFN-γ circuit for the molecular diagnosis of Mendelian susceptibility to mycobacterial disease. Crit Rev Clin Lab Sci. 2018 Apr 3;55(3):184–204. 10.1080/10408363.2018.1444580

2. Kratzer B, Grabmeier-Pfistershammer K, Trapin D, Körmöczi U, Rottal A, Feichter M, et al. Mycobacterium avium Complex Infections: Detailed Phenotypic and Functional Immunological Work-Up Is Required despite Genetic Analyses. Int Arch Allergy Immunol. 2023;184(9):914–31. 10.1159/000530844

3. Esteve-Solé A, Deyà-Martínez À, Teixidó I, Ricart E, Gompertz M, Torradeflot M, et al. Immunological Changes in Blood of Newborns Exposed to Anti-TNF-α during Pregnancy. Front Immunol. 2017 Sep 21;8(SEP). 10.3389/fimmu.2017.01123

4. Maecker HT, McCoy JP, Nussenblatt R. Standardizing immunophenotyping for the Human Immunology Project. Nat Rev Immunol. 2012 Mar 17;12(3):191–200. 10.1038/nri3158

5. Cossarizza A, Chang H, Radbruch A, Abrignani S, Addo R, Akdis M, et al. Guidelines for the use of flow cytometry and cell sorting in immunological studies (third edition). Eur J Immunol. 2021 Dec 7;51(12):2708–3145. 10.1002/eji.202170126

6. Azarsiz E, Karaca N, Ergun B, Durmuscan M, Kutukculer N, Aksu G. In vitro T lymphocyte proliferation by carboxyfluorescein diacetate succinimidyl ester method is helpful in diagnosing and managing primary immunodeficiencies. J Clin Lab Anal. 2018 Jan 6;32(1). 10.1002/jcla.22216

7. Terrén I, Orrantia A, Vitallé J, Zenarruzabeitia O, Borrego F. CFSE dilution to study human T and NK cell proliferation in vitro. In 2020. p. 239–55. 10.1016/bs.mie.2019.05.020

8. Types of Correlation: Positive & Negative Correlation | tastylive [Internet]. [cited 2023 Aug 16]. Available from: https://www.tastylive.com/concepts-strategies/correlation
